# What supports and services post COVID-19 do children with disabilities and their parents need and want, now and into the future?

**DOI:** 10.3389/fpubh.2024.1294340

**Published:** 2024-04-08

**Authors:** K. Pozniak, A. Swain, G. Currie, A. Doherty-Kirby, D. Grahovac, J. Lebsack, W. Campbell, C. Humphreys, S. Patterson, S. Raha, J. Whitley, O. Kraus de Camargo

**Affiliations:** ^1^CanChild Centre for Childhood Disability Research, McMaster University, Hamilton, ON, Canada; ^2^Department of Pediatrics, McMaster University, Hamilton, ON, Canada; ^3^School of Nursing and Midwifery, Mount Royal University, Calgary, AB, Canada; ^4^School of Rehabilitation Science, McMaster University, Hamilton, ON, Canada; ^5^Faculty of Education, University of Ottawa, Ottawa, ON, Canada

**Keywords:** childhood disability, parents, youth, children, COVID-19, qualitative research, health service access and utilization, education

## Abstract

**Introduction:**

Children and youth with disabilities and special healthcare needs, and their families, have been uniquely affected by the COVID-19 pandemic. However, the voices of children themselves are still not well represented in the existing literature.

**Methods:**

This qualitative descriptive study used a combination of visual methods and interviews to learn about the experiences of Canadian children with disabilities (n=18) and their parents (n=14) during the COVID pandemic and into the post-pandemic period. Data collection was carried out between January and July 2023. The aim was to identify the supports and services children and families need at present and moving forward.

**Results:**

Families’ pandemic experiences were complex and nuanced. For many, the pandemic complicated and disrupted everyday activities and supports. These disruptions were largely buffered by parents. However, some families also identified unexpected benefits. Key themes pertaining to present and future needs included the need for services that are flexible; consistent; conducive to relationship-building; comprehensive; coordinated across sectors; and designed to support the needs of the whole family.

**Discussion:**

Implications for policy and practice are outlined.

## Introduction

Children and youth with disabilities and special healthcare needs, and their families, have been uniquely affected by the COVID-19 pandemic. Many of these children require medical care, therapies, home-or school-based supports—which were either reduced, shifted to virtual delivery or outright canceled throughout the pandemic (1–8). Reductions in school and community-based programs and services impacted these children’s already-limited opportunities for participating in physical activity and social interaction, and created additional burdens and stress for parents, while also depriving them of essential support (2, 5, 8–12). Taken together, these changes have adversely affected children’s development, their physical and mental health and well-being, family functioning, and parents’ mental health (5–9, 11, 13–16). Conversely, families have also reported certain unanticipated positive consequences, such as more time spent together (17), the widespread adoption of virtual solutions in areas such as education or healthcare (3, 4, 18, 19), and reductions in stress for children for whom school was a stressor (20). As we move forward with post-pandemic recovery, it is imperative to draw on the lessons from the pandemic to identify children’s and families’ needs for services, as well as overall lessons for improving healthcare, education, and community support. This study explored children’s and parents’ reflections on their experiences during the COVID pandemic and into the post-pandemic period. We wanted to understand: (i) What were the gaps in services and support at school and in community services during the pandemic? and (ii) What supports and services do children and parents need and want, now and into the future? Our aim was to go beyond merely documenting the experiences of children and parents; rather, we wanted to identify what children and families need, at present and moving forward.

There is a substantial, and still growing, body of literature documenting the experiences of children with disabilities and their parents during the Covid pandemic. Studies from various settings across the world consistently show that the pandemic had a largely negative impact on these children and their families. Pandemic-related closures disrupted children’s routines (2, 11, 17, 21), and deprived them of needed supports such as therapies and recreational activities, as well as contact with peers and other important adults in their lives (2, 12). Overall, children had more screen time (11, 14, 17, 21), less physical activity (5, 11, 14, 22), poorer nutrition (11, 23), and poorer sleep (11, 24). Many children had difficulties with learning remotely, due to challenges with attention and focus (2, 4, 5, 7, 17, 23, 25). Although for some children the home environment was more conducive to learning than school (4, 17, 19), this required extensive parental support (2, 4, 17, 26). Furthermore, not all families had adequate access to technology needed for online learning (for example, high-speed and unlimited internet is unaffordable for some families and entirely unavailable in some remote and rural communities), which led to a deepening “digital divide” between families (4, 19, 23). Taken together, these experiences affected children’s emotional well-being, with many children experiencing and expressing stress, frustration, anger and anxiety (4, 5, 9, 11, 14, 23). Some children were also reported to regress in development and lose social skills (5, 9, 11, 21).

As most of the world shut down, an essential activity that had to continue was caregiving. It fell to parents and other caregivers to buffer the closures and disruptions of community and social supports such as childcare, respite care, nursing and personal support worker (PSW) care, and to support their children’s learning (2, 14). Parents struggled with having to juggle the physical and mental load entailed in these responsibilities (5, 8, 11, 12, 16, 17, 23, 26). Some parents—especially mothers of younger children—had scaled back, or entirely given up paid employment, which impacted their financial security (4, 8, 17, 23, 27). Taken together, the toll of filling the gaps in the social support system impacted parents’ mental health, resulting in stress, burnout, anxiety and depression (5, 9, 11, 12, 15–17, 23). For the most part, families did their best under the circumstances (4, 12). Many parents reported that their families had more time to spend together (17, 19), and some families benefited from virtual solutions for learning, healthcare appointments, or connecting with friends and family (2, 12, 19). However, not all families fared equally well, with some experiencing issues such as poverty, substance abuse, or domestic abuse and violence (4, 17, 23).

Many of the challenges described above were experienced by most children and families, and are not unique to the context of disability. However, children with disabilities and their families experienced a higher rate of these adverse events in comparison to the general population (8, 28). At the same time, many of the challenges experienced by families of disabled children during the pandemic were not new to them—rather, the pandemic exacerbated the pre-existing issues they were already facing (2–4, 12). For many families, the experience of inadequate social or financial support was in fact “nothing new” (3). In all, we can summarize that the experience of disability exacerbated the impacts of the pandemic, and the pandemic exacerbated pre-existing gaps and lack of prioritization of disabled people (3, 4, 25).

Children’s own perspectives and experiences are increasingly being recognized as important to informing research and policy. The United Nations Convention on the Rights of the Child (29) paved the way for a recognition of children as social agents who are knowledgeable about their lives, and who have a right to participate in making decisions on matters that affect them. In effect, there has been a shift from doing research *on* children (where children are the objects of research, but their experiences are represented by others—for example their parents), to doing research *with* children, and increasingly to research done *by* children (in which children shape the research agenda as researchers) (30–32).

As children’s involvement in research is becoming more prevalent, researchers are drawing on various (often non-traditional) approaches that allow children to express themselves in ways that best work for them. One approach is the use of arts-based methods such as drama, dance, or visual methods using drawings, photos, or filmmaking (33–35). These methods are seen as child-friendly as they are more familiar (and often enjoyable), and allow children more agency in expressing themselves (36, 37).

Although more and more research is being done *with* and *by* children, a recent review found that children with disabilities are still not very well represented in research studies (30). Leaving out their perspectives is problematic, as it contributes to their societal exclusion (30). There are notable exceptions to this, with important work being done to incorporate disabled children’s perspectives, and to involve them in the research process (38, 39). Concomitantly, new guidelines and best practices have been developed regarding the ethical considerations entailed in doing this work (40), and methods for engaging children who cannot self-express in traditional ways (41–43). Our research study sought to build on this work by: (i) seeking the perspectives and opinions of disabled Canadian children and youth as well as their parents; and (ii) engaging a group of disabled youth as research collaborators in designing, carrying out and disseminating research.

## Materials and methods

### Conceptual framework

Conceptually, this study is informed by the World Health Organization’s International Classification of Functioning, Disability and Health (ICF) (44), and specifically its translation into the concept of the F-words for Child Development. This framework describes the various domains of life that influence health (family; friends; fun; functioning; fitness; and future), and the interrelationship between them (45). It provides a holistic lens to capture the multiple factors affecting children’s and families’ well-being and identify intersections across various life contexts. Children with disabilities receive supports and services in many settings, including healthcare and education. However, despite the multiple overlaps between these domains (for example, therapies delivered at schools), researchers and practitioners from these two sectors rarely work together. In this study, we aimed to explicitly bridge these siloes by identifying themes in experiences and lessons that cut across systems and sectors.

### The process: integrated knowledge translation

The study design is informed by principles of patient-oriented research as outlined in the Strategy for Patient-Oriented Research (SPOR) by the Canadian Institutes of Health Research (46). Our team used an integrated knowledge translation (iKT) process, according to which researchers and knowledge users (in our case, parents and children/youth) collaborate across all stages of the research process, from planning the study to disseminating the results. Four parents are co-investigators on the study (one of whom is also the co-PI), and four of their children are collaborators or “junior researchers.” The study received ethics approval from McMaster University’s ethics board (#15157).

The parent partners have been part of the team since the grant-writing stage, and have been involved in all team meetings, and in making all decisions related to the progress of the study. All of the parents have extensive experience and training in Family Engagement in Research (FER). In addition, one of the parents (GC) instructs a program in FER at our institution, and another parent (ADK) runs the @youth_in_research Instagram account for youth who are involved in research. The parents have guided the team on how to work together with them and their children (the junior researchers). Together we have developed recruitment materials and study instruments. The parents also read through the transcripts, advised on the generation of themes, and provided feedback on subsequent versions of this article. Three of our parents (DG, ADK and JC) have backgrounds in various aspects of knowledge translation (including design, web design, and social media) and are advising us on our knowledge translation strategy for different audiences.

The four youth collaborators are involved in the study in advisory roles. When the study began they were between 10 and 19 years old, and live with various diagnoses including cerebral palsy, ADHD, learning challenges and rare conditions. They have been part of the team since the grant-writing stage, at which time they were introduced to the study and expressed their interest in being involved. With their parents’ support as needed, the youth attend small-group meetings, facilitated by KP and AS. They have advised us on the development of recruitment materials (one of them assisted with recruitment by contributing material to the creation of a recruitment video) and research instruments, to make sure that they are appealing, meaningful and accessible to youth. They reviewed some of the data (namely, the completed Time Capsule worksheets), and are currently advising us on our knowledge translation strategy.

### Participants and recruitment

Children/youth with disabilities and/or additional healthcare needs, along with their parents/caregivers, were invited to participate in the study. This approach aligns with family-centered approaches in child health, which focus on the family (rather than on parents or children individually) as a unit of attention (47). Our group embraces a non-categorical model of disability (48), so participation was open to any child with a disability regardless of diagnosis. Because we were particularly interested in children’s and families’ experiences and needs as they relate to healthcare and education sectors (and the intersections between these sectors), we sought to engage children who were school-age at the time of pandemic lockdowns in 2020, and who were still in school when the interviews were conducted between January and July 2023. Thus we recruited children between the ages of 8 and 21, since in some Canadian provinces children with disabilities can attend high school until the age of 21. Participants were recruited through our research center’s social media networks, including our online newsletter, closed Facebook group for parents, as well as through the social media networks of our parent investigators (including the @youth in research Instagram account).

Participants who expressed interest in the study (in our case, the person who initiated contact with us was always the parent), were invited to attend a video call along with their child(ren), during which two researchers introduced themselves, told the youth about the study by sharing slides with images, and answered any questions they had. At the end of the video call the youth were asked whether or not they were interested in proceeding with the study. We also explored with the youth and parents what accommodations they might need in order to facilitate their participation. If the youth expressed willingness to proceed, we e-mailed the parents the official consent and assent forms for them and their child(ren) to complete. If the youth were able to independently sign the assent form, they signed it themselves and the parents returned it to us via e-mail. If they were not able to sign, the parent signed on their behalf and returned it to us along with their own consent form. When we met again for the interview, we always began by asking the youth whether they were still interested in talking with us about their pictures. We checked with them throughout the interview and offered breaks or the opportunity to end and continue at another time if they wished.

### Study design

This article reports on the first phase of an exploratory sequential research study, in which qualitative methods are being used to inform the development of a survey (49, 50). The methods used in this phase were intentionally selected to be child and youth-friendly, fun, and flexible, in order to allow all interested children (including children who use communication assistive devices, or children with sensory sensitivities) to participate in a way that worked for them. This approach recognizes that “research with children demands flexibility and creativity on the part of both researchers and their ‘data collection’ approaches,” and hence flexibility is an important element of doing research with children (42, 51). The study methods consisted of (i) completing a visual worksheet that (ii) informed subsequent qualitative interviews with children, followed by (iii) interviews with their parents (Supplementary File 1). Although our research protocol allowed for flexibility within this research design (for example, the visual component was optional, and we were open to adjusting the interview component depending on participants’ needs and preferences), all of the participants completed all steps as originally designed with minimal adjustments (for example, one participant’s interview was split between two sessions to ease the burden).

(i) *Visual worksheet*. Art-based research (using drawing, painting, photography or drama) is a research method often used in research with children and/or individuals who have difficulties with speech production, reading or writing (10, 30, 34, 52). Visual tools provide an alternate way for participants to express their experiences. They can also be used as prompts for facilitating conversation and reflection during interviews (52, 53), and can be used alongside interviews to enhance and triangulate the data (52, 54, 55). They also help build rapport and minimize power differences between the researcher and the participant (52). Lastly, because visual outputs are typically more accessible to non-academic audiences than traditional research outputs such as publications, they can support the dissemination of research findings (52).

Child/youth participants were invited to complete a visual worksheet called the “Covid Time Capsule” (Supplementary File 2) on their own time. The Time Capsule asked them to either draw or electronically paste images that represented their experiences in various areas of life. These domains (Family, School, Fun, Fitness, Friends, Future) are informed by the ICF framework (44) and in particular its translation into a framework called the F-words framework (45). Participating children/youth were able to complete the worksheet either in hard-copy by hand (by printing it out) or by pasting images into a form-fillable file. The children customized these options in ways that worked best for them, often combining their own photos, images from the internet, hand-drawn pictures, and text (whether typed or hand-written). One child made her own hard-copy collages, took photos of them, and pasted those images onto the worksheet, while another child disregarded our template altogether and created her own slides from scratch.

Depending on their age and situation, the children/youth could complete the Time Capsule independently or with parent support. The majority of youth (with the exception of six) received some degree of parental support: for example, they chose the images and their parents helped paste them into the form-fillable document.

(ii) *Interviews with children*. After completing the Time Capsule, the children/youth were invited to take part in a qualitative interview that used their images as a springboard for exploring their experiences during Covid and their needs for services and supports. The interview questions were flexible, based on the images provided by the participants. During the interview, the interviewers went through each participant’s Time Capsule page by page and asked questions about the images. For example, participants were asked about the activities depicted in the images (e.g., playing a sport), how often the child participates in them, and whether and how their participation was impacted by the pandemic.

All of the interviews were conducted virtually using videoconferencing software. Children/youth could take part in the interview alone, or with a parent or support person of their choice. Eleven of our child/youth participants had their parents present during the interview. Parents who accompanied their children typically sat back (sometimes off-camera) and did not interject except when the child did not hear or understand a question, or sometimes to provide clarification or elaborate on the child’s response. Four of the children interviewed needed extensive parental support: two of those children needed the parent to repeat questions and help them stay on task when they got distracted; one child communicated with a speech device which his mother helped him navigate; one had a language delay and needed their mother to provide prompts to the questions and to elaborate/provide context to responses.

(iii) *Interviews with parents*. Following the child/youth’s interview, their parents took part in a separate interview at a later time. These interviews were semi-structured and explored issues such as: the pandemic’s impact on the parents, their children and families; the challenges and facilitators they experienced; their needs for supports and services; and suggestions for service delivery in the post-pandemic period. Parents also provided additional context to the images and reflections contributed by the youth.

Interviews were conducted by KP (researcher) and AS (research coordinator) together, with the exception of four interviews that were conducted by AS alone. Child/youth interviews lasted approximately 30 min, and parent interviews lasted approximately 60 min, although a few extended up to 90 min. In the case of the participant who used a speech device, the interview took approximately twice as long, and we divided it into two separate sessions so as to not over-tire them. All interviews were recorded and then transcribed by a professional transcriptionist.

### Analysis and interpretation

A qualitative descriptive approach (56–61) was chosen for this study. This approach aligns with our goal to describe and document participants’ perspectives on their experiences. It aims to capture the “who, what and where of events” (56) allows researchers to stay close to the data; and does not require an abstract rendering of the data in terms of a conceptual or theoretical framework. Instead, researchers aim to produce a representation and interpretation that the participants themselves would agree is accurate. The ultimate goal is to generate findings that will be useful to practitioners and policy makers.

The analysis process consisted of two steps: (i) content analysis of Covid Time Capsules; and (ii) thematic analysis of interview recordings. The Covid Time Capsules were analyzed using conventional content analysis, which allowed us to identify the content in children’s images (e.g., playing sports, presence of friends or other significant “helpers”). The interviews were analyzed using reflexive thematic analysis (62, 63), a type of thematic analysis often used within constructionist, relativist and/or critical realist approaches. Using this approach allowed us to inductively identify latent patterns of meaning pertaining to participants’ experiences that went beyond surface content (for example, the need for flexibility in services, or the importance of continuity in relationships). Since reflexive thematic analysis treats researcher subjectivity as a resource to knowledge production (rather than as a threat to “objectivity”), we were able to draw on our knowledge and experiences to connect the gaps and needs identified by participants to the types of services and supports that would address these needs.

The initial analysis was carried out by KP and AS. KP and AS reviewed and made notes on the content of each Covid Time Capsule, then read and coded each set of family transcripts (first the child’s, then the parent’s) using an open coding approach. They met after coding each transcript to compare and discuss codes and to collaboratively develop a codebook, which evolved as new codes were added based on new data. Although we allowed the codes to emerge inductively, our thinking was informed by the conceptual model of the F-words that informed our study, as well as by the design of the Covid Time Capsule worksheet which directed us to pay attention to the domains of life captured by the F-words framework (family, friends, fun, fitness, future and school). However, we also recognized multiple overlaps between these domains. For example, some children had *fun* playing sports (“*fitness*”) with *friends* at *school*—a finding which is consistent with the ICF framework’s tenet that these domains are interrelated. Therefore, our final thematic structure sought to identify patterns in the data that cut across domains. We explored patterns both between and across categories; that is, we analyzed children’s Time Capsules and transcripts in relation to those of other children; parent transcripts in relation to other parents; and the entire “family package” of child and parent transcripts in relation to those of other families. The remaining members of the study team also read a selection of transcripts and identified key patterns and messages in the data. The research team held three meetings to discuss emerging themes from the transcripts, as well as contributed feedback asynchronously.

### Reflexivity

In accordance with the tenets of thematic analysis, we reflected on how our disciplinary and personal backgrounds informed our engagement with the data. KP is a socio-cultural anthropologist whose research focuses on the experiences of parents of children with disabilities, as well as a mother of two elementary school-age children, one of whom lives with cerebral palsy. AS is the study coordinator with a background in clinical developmental psychology and professional interest in family well-being. GC is a nursing researcher whose work focuses on the experiences of parents caring for children with rare conditions, as well as a mother of two teenage boys with disabilities. ADK, DG and JL are disability advocates and mothers of children with disabilities. OKDC is a developmental pediatrician with an interest in applying the ICF framework to health services. WC is a speech-language pathologist whose research focuses on inclusive models of service delivery in schools. SP and CH are both child life specialists. SR is a biochemist as well as the co-founder of our university‘s Children and Youth University which delivers free science programs to children. JW is an education researcher whose work focuses on inclusive education preparation, policy and practice.

This diversity of backgrounds and experiences shaped the questions we posed, and the themes we identified in the data. The mothers among us experienced firsthand the toll of juggling the demands of working and caregiving during the pandemic, as well as the pandemic’s detrimental impact on our own children and family lives. The pediatric clinicians were similarly concerned about the pandemic’s impact on children, as well as keen to explore the perspectives of children. The education researchers were interested in the school-related experiences of children with disabilities.

Throughout the research process we remained mindful of the power relations inherent in the research process as well as the nature of knowledge produced through the research encounter, which we recognize is always partial and co-produced through the interaction between the researcher and the participant (64). While these are important considerations in all qualitative research, they are particularly salient in research with children (65). In recent years, a number of researchers have cautioned against the risk of taking children’s voices at face value, and instead encourage researchers to recognize that children’s voices are always co-constructed by children and other adults and institutions with whom they interact (e.g., parents, schools), and thus informed by adult-child power relations (65). When we talked with youth participants, we attempted to be aware of these issues by explicitly reassuring the participants that “there are no right or wrong answers—we just want to know what you think,” and we attempted to follow their lead as much as possible by respecting their silences or wishes to explore certain topics over others. Although we recognize that there is no perfect way to “give voice” to research participants (since ultimately it is always the research team that controls the ways in which participants’ experiences are represented), we did our best to enable the children/youth to convey their experiences in a way that worked for them.

### Trustworthiness

The research team employed several measures to ensure trustworthiness during data collection and analysis. During the analysis stage, the diverse backgrounds of the research team facilitated triangulation. Parent investigators contributed significantly to theme development by identifying themes in the transcripts that resonated with their own experiences as well as the experiences of other parents they know through their multiple networks. Other members of the research team similarly confirmed that the themes generated by us resonate with the accounts they hear from other parents in their clinical and/or research work.

## Results

Altogether 14 families from 2 Canadian provinces took part in the research study. This included 14 parents and 18 children (four families had more than one child participate). Almost all of the participating parents were mothers, with the exception of one father. Participating children ranged in age from 8 to 16, and included 11 females and 6 males. The children/youth in the study had a wide range of diagnoses, including Autism Spectrum Disorder, Attention Deficit Hyperactivity Disorder, learning disabilities (e.g., dyslexia), autoimmune conditions as well as other rare conditions. Parent and child demographics are summarized in Table 1.

**Table 1:** Characteristics of parents and households.

Variable (Number of respondents)	Category	Frequency	Mean (SD) Range
Parent background information (*n* = 14)			
Age (years)			45.14 (5.95) Min = 36, max = 54
Gender	Female	13 (92.9%)	
	Male	1 (7.1%)		
	Non-binary/Other	0		
Relationship to child	Mother	13 (92.9%)		
	Father	1 (7.1%)		
Province of residence	Ontario	13 (92.9%)		
	Alberta	1 (7.1%)		
Type of community	Large city/urban area (>500,000 people)	9 (64.3%)		
	Small or medium sized town (100,000–500,000 people)	2 (14.3%)		
	Rural area	3 (21.4%)		
	Other	0 (0%)		
Current employment status (*n* = 14; not discrete: parents could indicate more than one option)	Full-time	5 (35.7%)		
	Part-time	4 (28.6%)		
	Not in the paid workforce right now	3 (21.4%)		
	Volunteering	1 (7.1%)		
	Full-time caregiver	7 (50%)		
	Other (self-employed)	1 (7.1%)		
First language	English	11 (78.6%)		
	Other (Russian, Assyrian, Spanish, French)	3 (21.4%)		
Birth location	Canada	12 (85.7%)		
	Other (Russia, Mexico)	2 (14.3%)		
Ethnicity	Black	1 (7.1%)		
	Hispanic	1(7.1%)		
	White/Caucasian/European	9 (64.3%)		
	Mixed	2 (14.3%)		
	Other (prefer not to answer)	1 (7.1%)		
Household Income	<$25 k	4 (28.6%)		
	$25– < $50 k	1 (7.1%)		
	$50– < $70 k	1 (7.1%)		
	$70 k– < $100 k	1 (7.1%)		
	$100– < $150 k	3 (21.4%)		
	>$150 k	4 (28.6%)		
Number of children in the family, total (*n* = 25)	1	3 (21.4%)	
	2	11 (78.6%)	
Age of all children in the family (*n* = 25)			12.68 (3.46) Min = 3, max = 18
Number of children *with diagnoses* in the family (*n* = 20)	1	8 (57.1%)	
	2	6 (42.9%)	
Age of children *with diagnoses* in the family (*n* = 20)			12.8 (2.75) Min = 8, max = 18
Child diagnoses (not discrete or exclusive)* (*n* = 20)	ASD	9	
	ADHD	9		
	Learning disability (including dyslexia, dysgraphia, dyscalculia)	10		
	Other neurodevelopmental/ neurological disorders	6		
	Mental health diagnosis (e.g., anxiety disorders, OCD, BD, PTSD)	10		
	Overall developmental delay/impairment	5		
	Neuromuscular disorders	1		
	Behavioral/emotion regulation difficulties	1		
	Other (15q26 chromosome deletion, cloacal malformation, osteopenia, autoimmune condition)	4		
Health services *any child in the family* received (not discrete—parents could indicate more than one option) (*n* = 20)	Speech therapy	9	
	Occupational therapy	11		
	Physiotherapy	4		
	Behavior therapy	6		
	Medical services (doctors and surgeons)	9		
	Other (psychotherapy and mental health support, specific skills or wellbeing coaching/training, RMT)	4		
Household composition (not discrete—parents could indicate more than one option)	Spouse/Partner	9 (64.3%)		
	No one	5 (35.7%)		
	Other (e.g., aunt, grandparents)	0		
Caregiving support (not discrete—parents could indicate more than one option)	Spouse/partner/parent of child	7		
	Grandparent	4		
	Other (e.g., other family member, neighbor, personal support worker)	4		
	No one	3		

In general, the accounts of children and their parents complemented and enhanced each other. Parents provided longer and more detailed responses than their children, and, since the parent interviews took place after the children’s, parents were able to provide additional context into some of the topics raised by their children. In a few instances, the parents remarked that seeing their children’s Time Capsules, or hearing their interviews, gave them new insights into their children they did not have before; the most strident example of this was a mother whose son told us in the interview that he did not like any of his nurses or personal support workers. Parents were also more likely to describe challenges in the healthcare, education, and social support systems.

Below we present parents’ and children’s insights as they relate to two interrelated topics: Families’ experiences during the pandemic; and What families need to move forward and thrive, now and into the future. A summary of themes and subthemes can be found in Table 2. All the participant names used throughout the article are pseudonyms.

**Table 2:** Characteristics of child participants.

Variable (Number of respondents)	Category	Frequency	Mean (SD) Range
Child Background Information (*n* = 18)			
Age (years)			12.67 (2.52) Min = 8, max = 16
Biological sex	Female	11 (61.1%)	
	Male	7 (38.9%)	
	Non-binary/Other	0	
Child diagnoses (not discrete or exclusive) (*n* = 18)	ASD	8	
	ADHD	9	
	Learning disability (including dyslexia, dysgraphia, dyscalculia)	8	
	Other neurodevelopmental/ neurological disorders	5	
	Mental health diagnosis (e.g., anxiety disorders, OCD, PTSD)	8	
	Overall developmental delay/impairment	3	
	Neuromuscular disorder	1	
	Behavioral/emotion regulation difficulties	1	
	Other (15q26 chromosome deletion, cloacal malformation, osteopenia, autoimmune condition)	4	

## Families’ pandemic experiences were varied and complex

In all, families’ experiences during Covid were varied and complex. Families described coping with disruption and loss, although they also remarked on certain unanticipated positive outcomes from the pandemic. The gaps in services and supports were filled by parents, often at a great cost to them. While every family’s experience was different (and in fact sometimes even children in the same family had different experiences), taken together the families’ accounts highlight certain key patterns pertaining to their experiences during the pandemic, and their needs for services and supports moving forward.

### Covid complicated and disrupted everyday activities and supports

**Figure 1:**
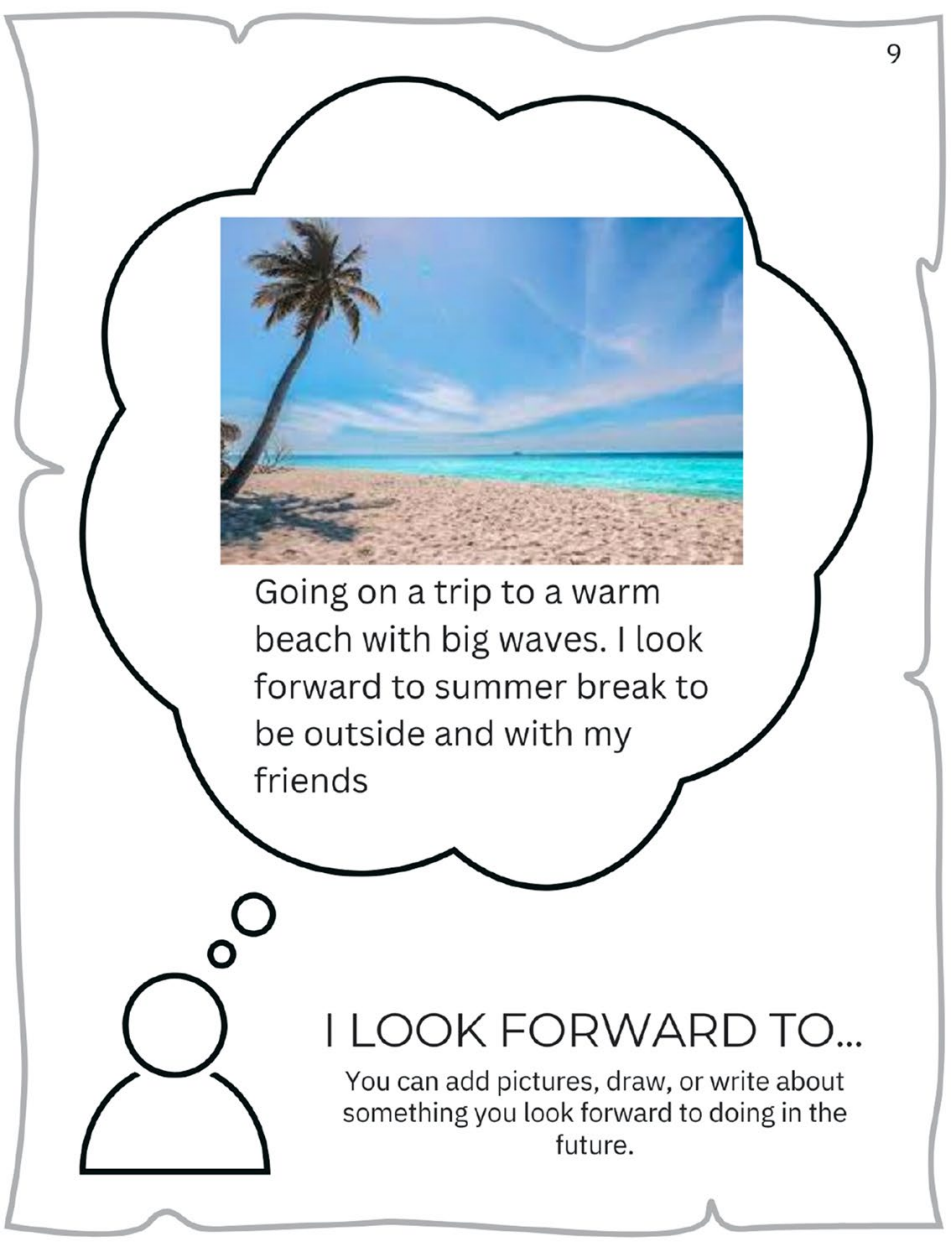
‘Eric’, 8 y.o., missed going on trips with his family.

**Figure 2:**
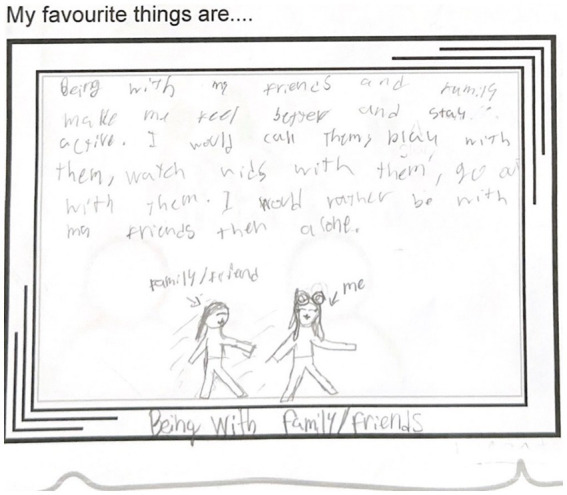
‘Nickie’, 12 y.o, missed spending time with her friends.

For many families, Covid complicated and disrupted everyday activities and supports. Several parents spoke about missed or delayed medical appointments due to Covid-related cancelations, which in some cases resulted in medical issues being missed. Participants also missed out on visits and interactions with friends and extended family members (e.g., grandparents), regular recreation activities (e.g., sports), and traveling (Figure 1). In-person school closures resulted in missed opportunities for recreation and social activities (e.g., spending time with friends at lunch or recess). Nickie (12 y.o.) talked to us about how important her friends were to her, and how much she missed them during the lockdown:

“Being with my friends and family makes me feel better and stay active. I would call them, play with them, watch vids with them, go out with them. I would rather be with my friends than alone.” (Figure 2).

When in-person learning resumed, many school activities (e.g., extracurriculars) were either canceled or had extra layers of restrictions imposed on them. Many of the children/youth in the study described their experiences during the lockdown period as “boring.” Many parents observed that their children regressed in learning and other skills due to the closure of in-person schools and therapies, and described their children as “lost,” “forgotten” or “falling through the cracks.” One such parent was Kora, mother of two children who had drastically different experiences with virtual learning. This is how she described her son:

“He was forgotten for two years. All of his therapies ended. There was no education, he could not do online, so there was no education, there was no interaction, there was no respite, there was nothing. It was me and him…. What happened was, when everybody went back, he had aged out of certain programs. We lost Speech altogether. Now we are waitlisted to get school-based language support. Speech was his number one priority of all his therapies, and we lost all—and during ages of seven, eight, nine, there was so much that he needed and that’s when we lost everything. It increased his sensory and increased his—he really struggled with transitions because of that. It was terrible…He fell through the cracks” (Kora, mother of two).

Parents, as well as some children, noted the mental health impact of isolation. Kyla, for example, described that her then eight-year old son was “crying himself to sleep” as a result of his struggles with online learning: he worried that he will never be able to graduate school and get a job.

Parents also spoke about losing nursing and homecare supports due to the overall shortage of workers, an issue which continues to persist. Parents noted the “revolving door” of workers, as well as the lack of professionalism among the small number of workers who were available. Kora, for example, described experiencing theft and substance abuse from her child’s respite workers.

We have a serious lack of respite in our city. They’re offering to pay to train people through our Child Development Centre to become respite workers…. We’ve had two workers end up being charged criminally, we have had one here that was wasted, we had one steal a ton of money from us. Enough that [my husband] quit his job (Kora, mother of two).

Lilly, who managed a staff of seven part-time nurses and three part-time PSWs for her medically complex child, similarly reflected on the difficulties with finding and retaining workers, and the implications of this on continuity of care:

All of my nurses work somewhere else, because you can never get anybody full-time, they are all working at hospitals … they are all just sort of doing part-time in my house. I basically have to take what I can get. So yes, that’s why the staff is so hard and it becomes very convoluted and hard to make sure that people are—the continuity of care, you know, if something changes making sure everybody is updated and knows. Like [my son] has a broken leg. One of my PSWs came in the other day and she’s like ‘oh, he does’? I’m like ‘his leg has been broken for two weeks and you do not even know yet’?

**Figure 3:**
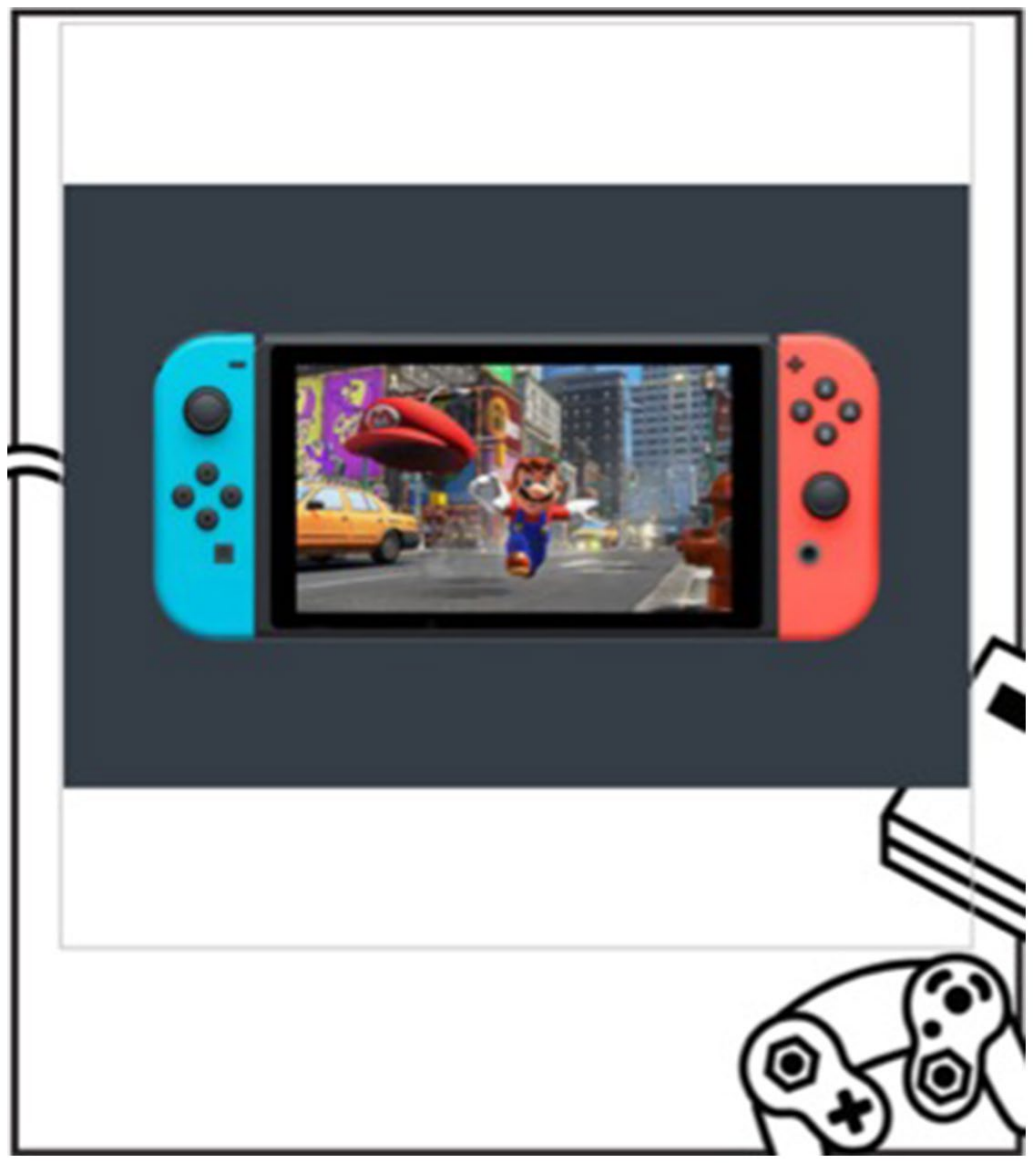
Many children included images of video games. During the pandemic this was a common way for children to connect with friends (‘Eric’, 8 y.o.).

Families coped with the closures, restrictions and losses, as well as they could. Families spent a lot of time together doing activities such as going for walks, cooking or baking, or playing board games. Children spent more time on computer screens, whether on social media or playing video games (Figure 3). Most parents attempted to maintain some extracurricular activities for their children in a virtual format (e.g., music, dance, karate, theater), and some children received some of their therapies virtually. A few older children reported coming up with their own coping strategies, such as developing an exercise schedule.

### Covid was a “mixed blessing”

Alongside stories of disruption and loss were also accounts of occasional unexpected “silver linings” that allowed for what was previously deemed impossible. One frequently cited example was the use of technology (especially virtual platforms) in school/education, healthcare, working, socializing and shopping.

**Figure 4:**
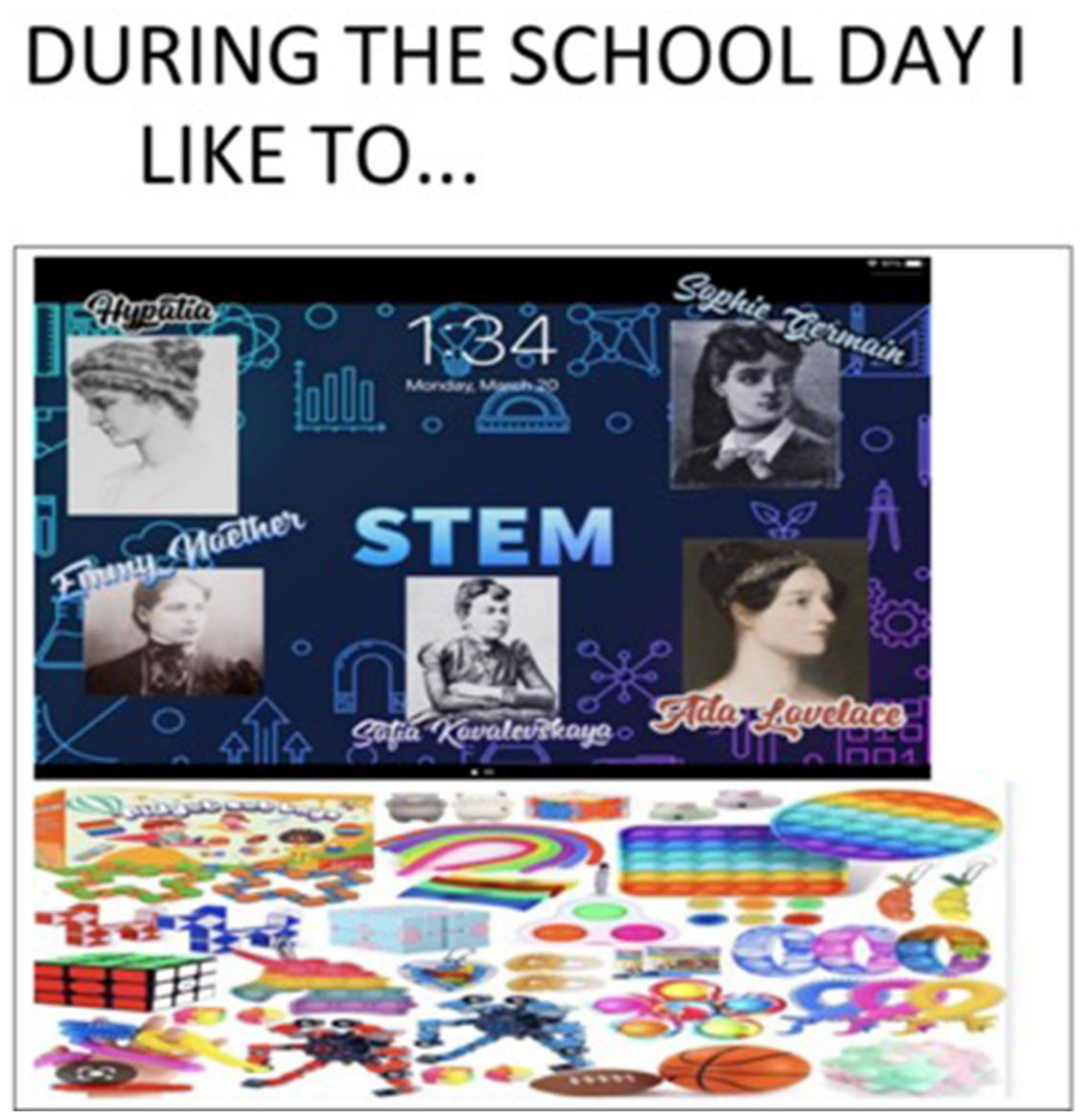
For ‘Michelle’, 13. y.o., virtual learning was an opportunity to discover her strengths and excel in her studies “The picture is my screensaver on my iPad. I’m in love with math and science so it’s STEM… and those are some famous women in math who started paving the way. The bottom picture is fidgeting because that really helps me have control during the day.” (‘Michelle’, 13 y. o.).

Education was identified as the key area where virtual solutions—when used in accordance with the child’s need, rather than imposed as a blanket “one size fits all” model—can be beneficial. Four of the parents indicated that their children benefited from virtual learning. Kora, whose earlier account described her son as “forgotten” during Covid, noted that for her daughter Michelle, Covid had been “a blessing.” For Michelle, a virtual mode of learning removed all the distractions associated with in-person schooling, which allowed her to discover her strengths and abilities. This is how Michelle described her experience:

“I like really found myself I’d say through Covid because it gave me a lot of time to figure myself out and figure my abilities out and figure my interests out, so if Covid did not happen I do not know if this would have happened, I really do not know. Because in in-person class I wasn’t the best, and then online class happened and I really had more time to focus on these things and it really helped” (Michelle, youth) (Figure 4).

In addition to virtual learning, parents also noted the benefits conferred by other virtual solutions. This included virtual healthcare navigation and appointments (particularly for parents who lived in remote locations), family therapy, and also online shopping (especially for parents whose children had difficulty in stores and public places). One girl who had been homeschooled prior to the pandemic benefited from a burgeoning of virtual programs which allowed her to explore new interests, and noted the benefit of virtual auditions for her dance program. Two parents were able to complete Master’s degrees virtually during the pandemic, and one of them secured a remote job that likely would not have been offered in a remote format prior to the pandemic.

Parents also noted other unexpected benefits from the pandemic. For two families, lockdowns ironically presented an opportunity for their children to make new friends, because their children were allowed to play outside with the other children in the neighborhood. Another mother noted that the loneliness and isolation her son experienced motivated him to become more social when schools reopened. Another mother shared that her daughter’s chronic pain seemed to have improved in the first month of the pandemic, a fact that the mother attributed to reduced pressure and stress from various activities and therapies (however, a different mother noted the opposite of her child). Two mothers mentioned that they benefited from their husbands being home more often to oversee the children’s online learning and to carry out home renovations.

In all, virtual healthcare, education and extracurricular programs created new possibilities for some participants. However, these benefits were variable and uneven: for example, children’s experiences with virtual schooling was highly dependent on their needs and personality. Nonetheless, these “silver linings” illustrate that, in retrospect, the supports that existed pre-Covid were not working as well as they should have been. For example, the fact that virtual learning benefited some children suggests that these children were not getting the supports they needed in-person.

### Parents filled in systemic gaps in care, education and therapies

**Figure 5:**
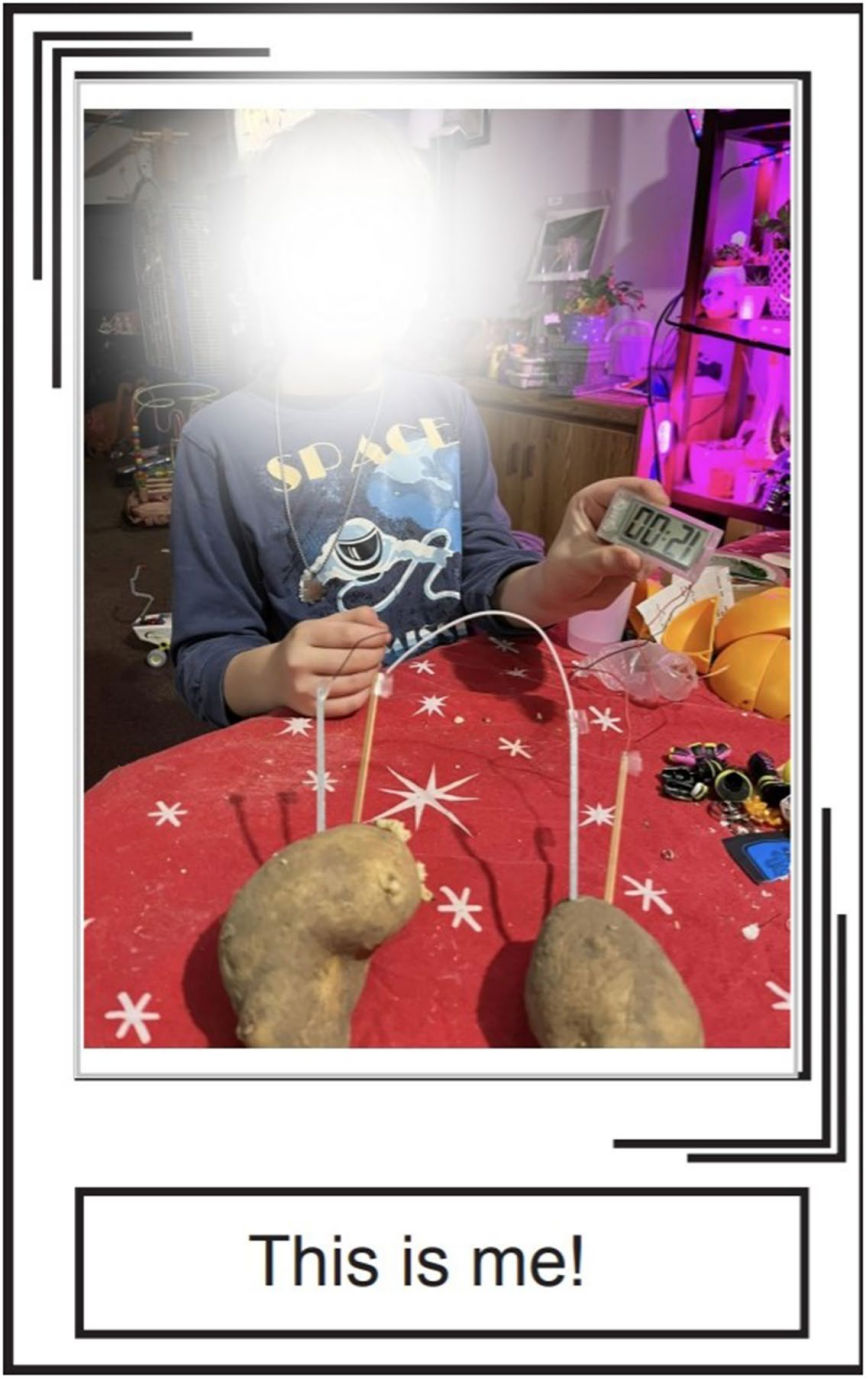
‘Ethan’, 8 y.o., made a potato clock with his Mom’s help.

Taken together, the accounts of parents and children described above illustrate the remarkable resilience of individuals and families. However, it must be noted that families’ successes were facilitated by the tremendous amount of work the parents invested in supporting their children’s development and well-being, including learning, therapy or recreation. When parents faced gaps in their support systems—whether in education, healthcare, childcare, or others—they had no choice but to assume other people’s roles, becoming teachers, therapists, nurses, care coordinators, and program planners, as needed (Figure 5). The burden of these roles became particularly visible during the pandemic, when supports such as schools, therapies, recreational programs and respite programs all shut down at once. Parents who worked from home since the beginning of the pandemic shared that remote jobs gave them more time to support their children’s needs, although these activities were also time-consuming and demanding. For example, Alex reported that the year of in-person school closures was a “mixed blessing” for his family. On the one hand, he had to severely scale down his work hours, which took a financial toll on the family. On the other hand, he was able to support his elementary-school age son’s learning and skill development. In effect, his son progressed at home much faster than he was doing at school, to the point that upon returning to in-person learning he no longer needed behavioral therapies. Alex reflected on this period as “one of the most magical experiences in my life. I miss it and I never want to do it again.”

**Figure 6:**
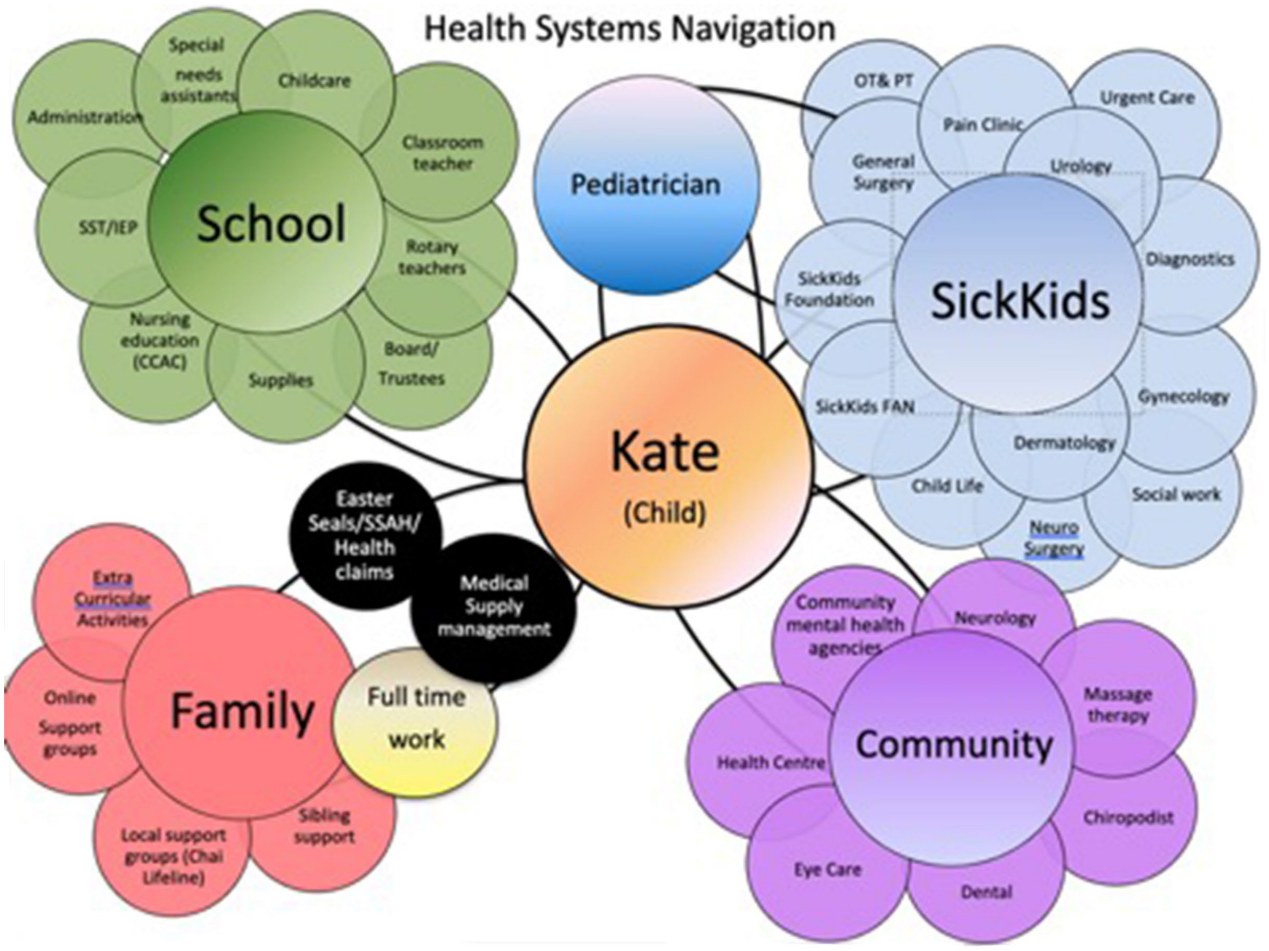
‘Meenah’, mother of two, shared a diagram of all the services she coordinates for her daughter.

While Covid-related closures were unprecedented, parents’ accounts illustrated that they were regularly expected to be their children’s therapists, nurses, and care coordinators, even during “normal” times. A few parents referred to the task of researching, advocating for, and co-ordinating, school-based and health services as “another job” (Figure 6). Many of the parents in the study described undertaking actions that would be considered as going “above and beyond” everyday parenting duties. For example, three of the mothers in this study had home-schooled their children, even outside of the Covid-related school closures (with one child returning to in-person learning). One mother spent hours every week on bookkeeping in order to reconcile funding for her child’s 10 different support workers whose compensation came from different funding sources (depending on whether the worker supported the child at home or school). A few parents engaged in advocacy that escalated to provincial ministries or human rights commissions. One mother (who had no healthcare background) obtained a job at her local children’s hospital so that she could better oversee her daughter’s care. She described her actions as a necessity:

“I’m going to do what I need to do because [child] would be dead. I know 100 % she’d be dead if I did not understand the system properly. Because we have had so many near misses and so this is what it took” (Meenah, mother of two).

Parents also demonstrated extraordinary ingenuity and initiative in determining solutions to buffer the chronic gaps and shortages. They described “out of the box” solutions such as engaging high school students to fold laundry and make sandwiches in return for volunteer hours, or renting out a room in the house to international students for extra income and childcare. Parental activities also included organizing accessible programs for other children who did not fit into “mainstream” programming. Alex, for instance, worked with his town to design an accessible “swim and gym” program for children with sensory needs after realizing that there were no suitable programs for his son. Lilly worked with her local children’s rehabilitation center on developing an accessible reading program for children who used assistive communication devices.

For the most part, parents were reluctant to acknowledge the toll associated with the multiple roles they were performing. When the interviewers opened up the topic, parents described their activities in terms of doing what was best for their child. Parents indicated that their work was essential in order to preserve their child’s health and well-being in the face of failed structures of support, and they expressed appreciation and even gratitude for whatever support they received, even if this support was inadequate to meet their children’s needs. However, filling systemic gaps in care, education and therapies, came at a cost to their own lives and goals. Several parents in the study have had to either scale down their employment, or quit working altogether, in order to provide their children with the support they needed. Esther described it in these terms:

“Right now I’m basically working half-time. I’ve had to subcontract a lot and get in a lot of help and so it really does reduce the amount of money that I make and it reduces the kind of services I can provide and the output and also I’m a professional woman so that’s a strong part of my identity” (Esther, mother of two high-school age youth).

And lastly, parents noted that many of the challenges spurred by Covid were actually “nothing new” to them. In fact, they reflected that these challenges might have enlightened the general population to some of the struggles that families who have children with disabilities experience on an everyday basis. As Ruth put it, during Covid “everybody kind of got to be a little disabled in some way,” in the sense that everyday activities (e.g., shopping, going to school) all of a sudden became a lot more difficult: “Here’s all these different things that people were forced to do and I think why a lot of disabled families did not really find it that difficult is because like, oh, it’s just another week for us” (Ruth, mother of two elementary-school age children).

## What do families need moving forward?

Families provided suggestions on what they need as they move forward after the pandemic. In accordance with the ICF framework which informs this study, we draw out larger themes and patterns that cut across life domains. These big picture themes include flexibility; constant and reliable social connections/supports; and comprehensive “wraparound” solutions. Below we elucidate on what these might look like in particular contexts such as school or community.

### Flexible programs and solutions

A recurring theme in parents’ accounts was that structures of support—whether in education, recreation, healthcare, respite, or funding programs—were “rigid” and based on a “cookie cutter” approach. In effect, children and families who did not fit the mold slipped through the cracks. This appeared to be the case for most of the children in our study. Parents often alluded to their child “not fitting in a box,” for a variety of reasons, such as: having multiple diagnoses; having a less-frequently encountered condition such as an autoimmune condition; or having varying support needs over time. The need for flexibility based on individual child and family circumstances in designing supports was a theme that cut across different life domains: schools, recreational/community activities and programs, work arrangements, and social supports.

Parents noted that schools operate on a “one size fits all” model for all children. Although the children in our study who had identified and documented healthcare and/or learning needs were required to receive accommodations and specialized supports, many of these supports were fraught with bureaucracy, and thus they fell short of actually meeting children’s needs. Kora, for example, told us about her child being denied recess as punishment for misbehaving, when in fact his individual education plan stipulated that he should receive extra body breaks. Trish described her child being denied the use of the sensory room at school, because access to that room was limited to children who were enrolled in a special program (which Trish saw as an attempt by the school to “push” her child into that program). Lilly told us that her child (who uses a speech device) was not able to receive help from an education assistant who was familiar with speech devices, because that individual was assigned to a different classroom.

Parents also cited numerous examples of school-based therapies that existed “on paper” but in fact eligibility criteria were so rigid that they were not actually provided to their children. These included, for example, a child being denied speech and language therapy at school because the school-based speech-language therapist was only allowed to work on articulation goals, whereas the child also had goals related to language which needed to be addressed first. A similar example concerned the inability of community-based occupational therapists to work on goals that were deemed to be school-related—and vice versa. Parents also noted that the frequency of services often did not meet their child’s needs.

Parental accounts point to the need for schools to provide more flexibility in delivering education and other disability supports (e.g., therapy) to students. The experience of virtual learning, though not the appropriate solution for all children and under all circumstances, nonetheless showed that different ways of learning and interaction are possible. Numerous parents in the study noted the possibility of offering hybrid (e.g., a mix of virtual and in-person) learning to students, particularly for students who often had to miss in-person school due to health concerns. One youth, when asked about her perfect school arrangement, replied “For the learning part, I would want to be online. But for the social part, I would want to be in-person because I cannot make friends online.”

Parents’ accounts also illustrated the need to remove artificial barriers such as denying a child with an individual education plan access to a sensory room because they were not enrolled in the special needs program. One youth provided a positive example of a flexible accommodation from which she benefited: in her case, it was the possibility of going to the resource room to complete her schoolwork if the classroom was too loud and she had trouble concentrating, or if she needed extra sessions at lunch. She described her resource room teachers as “helpful,” and the resource room as a place where she could go for help as needed.

The children in our study participated in an impressively wide range of artistic, social and other recreational activities. Parents’ accounts illustrated the amount of work required in researching activities that would be the right “fit” for the child, and supporting the child’s participation as needed. However many parents also remarked on the difficulty entailed in finding activities that worked for their child, and where their child could receive proper accommodation. Parents cited examples of their child being deemed “too disabled” for certain activities and “too high functioning” for others, and the overall difficulty of finding programs that were flexible and had adequate numbers of staff who were willing to work with the child on their own terms and assist them as needed. For example, Kora noted that her son does not qualify for any summer camps in her area because he is not toilet-trained. On the other hand, Ruth, whose two children had explored numerous sports, music and other recreational activities over the years, described one successful example of a skating program where her son was allowed to progress at his own pace and take breaks as needed, with an appropriate balance of support and flexibility by the instructors.

“Kevin would not really do the lesson. But they were great because they would just let him skate around. And so what would happen is he would just skate around, do his own thing the whole time, and every once in a while he might look and see what some of the other kids are doing and give it a try here and there. Or the instructor might pull him aside every once in a while and say, ‘oh here, try this’. And so it worked very well for him because he will not follow a full lesson, but he will do little bits and pieces here and there. His progression was nowhere near what it would be for other kids going through the same amount of lessons but he learned to skate” (Ruth, mother).

Parents’ accounts also highlighted the need for flexibility in their own work arrangements. Several parents in the study noted that they have had to scale back or altogether quit their jobs due to their inflexible work arrangements which made it impossible for them to combine working with caregiving responsibilities. For example, Trish, a pediatric nurse who quit her job due to inability to reconcile her work schedule with her daughter’s medical appointments, described her situation in these terms:

“I think once I had to leave my position, it’s essentially impossible to get back into the workforce especially because of her needs, and so things like benefits or days off, like sick time, that kind of stuff does not begin until six months to a year later. Well we cannot pause health care for six months to a year, we have doctors appointments almost every week, and so if you are upfront about the flexibility you need, people are like ‘no, that does not work’.”

A recurring theme in parents’ accounts was the overall rigidity in various systems of social support including funding, or nursing and respite care. Parents repeatedly noted that criteria for eligibility and for use of funds are “cookie cutter” and do not recognize the complexity of real-life circumstances. For example, Kora relayed a story about her son who needed a new stroller because he otherwise refused to leave the house and was a flight risk on the street. However, her insurance refused to fund the cost of a new stroller because her son was mobile. She was also unable to access government funding for therapies because that funding was only available for registered services, and no registered therapists were available in the remote community where she lived. Ruth described the challenges entailed in receiving funding for services from her provincial government, and Meenah recounted the difficulties of receiving school-based nursing for her daughter. Her local support agency had placed a blanket freeze on nursing care for medically complex patients, and was not willing to assign a nurse who needed to be present for the first 2 weeks of school only, in order to train school staff.

### Social connections/supports that are constant and reliable

Another key message in both children’s and parents’ interviews was the importance of having consistent and reliable social connections and relationships. Children’s Time Capsules included many images of people whom children described as important people in their lives (for example, when asked about what they liked to do at school, many children talked about having lunch with their friends or playing with their friends). Some children had difficulties forging relationships at school and needed opportunities outside of school (for example, recreational programs), with appropriate supports to facilitate socialization with peers.

**Figure 7:**
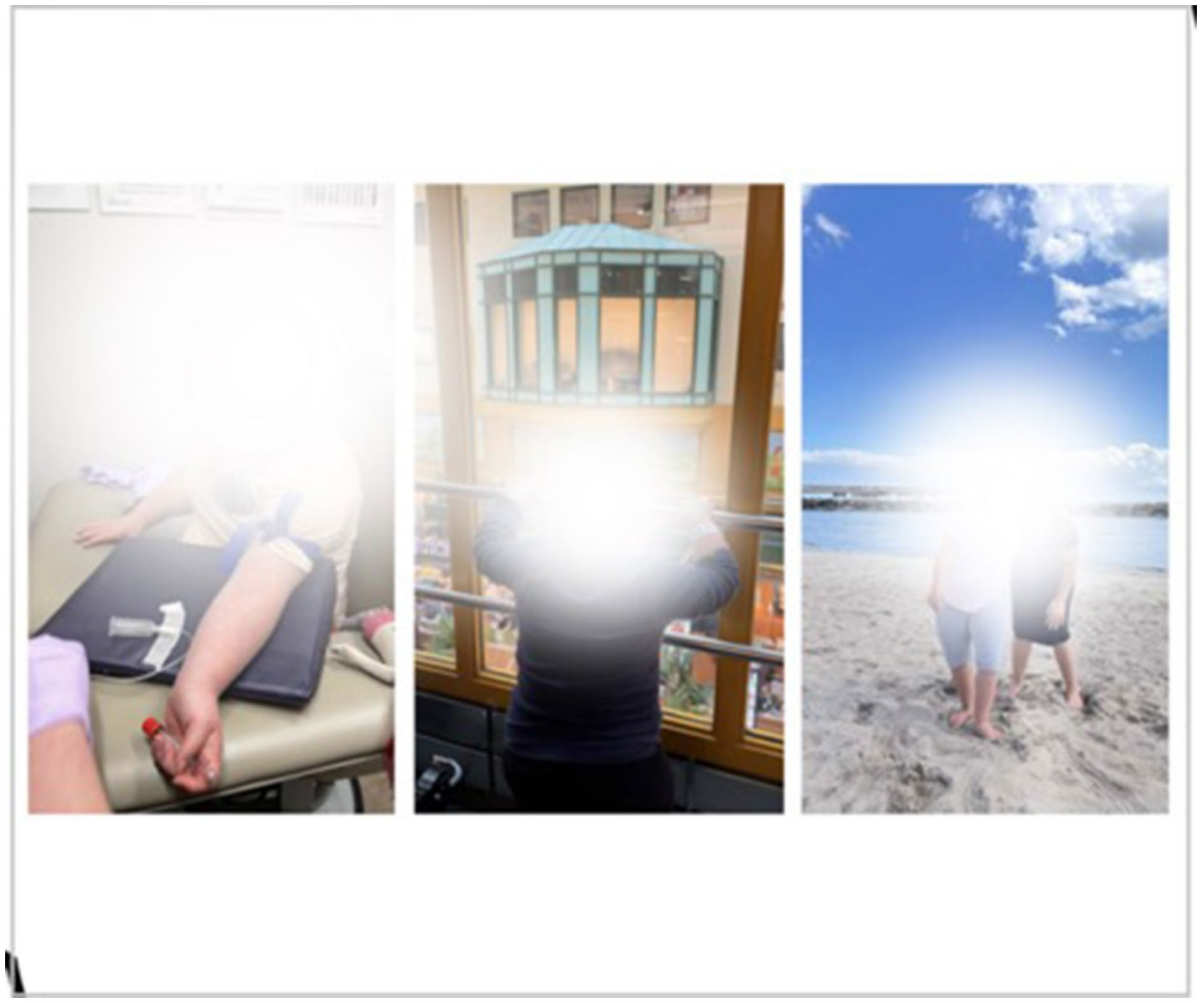
‘Zoey,” 14. y.o. enjoys traveling to her local children’s hospital to see her medical team and get her bloodwork done.

Besides peers, other special people mentioned in children’s Time Capsules and interviews included teachers, coaches, therapists, or doctors, with participants’ accounts showing the importance of having the “right people” who “click” with the child. For example, two children in the study talked about enjoying their regular hospital appointments on account of the good relationships they had with their medical team. One of these children even included a photo of having her blood drawn in her Time Capsule (Figure 7).

Missing out on social connections was one of the most frequently-cited repercussions of the Covid pandemic. In addition to missing contact with friends, both children and parents relayed accounts about important people who have moved on to different positions and locations. The pandemic exacerbated shortages of careworkers such as respite workers and school support staff such as educational assistants. In effect, parents lost needed helpers, and children lost relationships that were important to them. The high turnover of support workers made it difficult for children to build trust and maintain meaningful relationships. For example, Kora noted that her son experienced challenges in school due in part to the high turnover of education assistants: “You have a child who does not do well with change or transition or the unknown, needs routine, and you go to school and it was a different EA because the EA was constantly going on stress leave.” (Kora, mother of two). Similarly, Lilly’s son Connor told us that he does not like any of his ten nurses/support workers, a fact that Lilly attributed to the high turnover of care staff and their lack of time and interest in forging a meaningful connection with him. Participants’ accounts highlighted the need to cultivate meaningful opportunities for children to make and maintain friendships (at schools and community programs), as well as to have reliable and consistent relationships with important adults, such as careworkers, teachers, coaches and healthcare professionals.

Lastly, parents’ accounts illustrated the overall lack of caregiving support they received. None of the parents in the study received significant childcare assistance from informal sources (for example, from extended family such as grandparents). This was the case even prior to the pandemic; only one parent indicated that she received regular childcare support prior to the pandemic, which however was lost due to social distancing restrictions and never regained. Parents’ only sources of support were formal (school, respite workers, etc.), but these were insufficient, unreliable, and of low quality. As parents did their best to single-handedly fill in for an entire non-existing “village” of supports, they sacrificed their own personal and professional aspirations and well-being.

### Comprehensive and holistic “wraparound” supports for the entire family

Parents’ accounts indicated the need for comprehensive and holistic supports for the entire family. In particular, numerous parents noted that siblings of the child with a disability also needed attention and support. A few parents also noted that their own mental health and well-being was typically overlooked. One mother praised her daughter’s mental health intervention, which included a family component, for strengthening their family and helping them through challenging times.

Parents’ accounts illustrated that they desired—though rarely received—care that was coordinated and took into account the entire picture of the family’s life. Navigating and coordinating services took huge amounts of parents’ time and energy. For example, Meenah reported having as many as 80 individuals on her daughter’s care teams across different health organizations. Only two parents in the study received support with care coordination/navigation, and one of those parents noted that she still had to seek out her service navigator herself in the first place. Lilly, whose medically complex son was part of a complex care team, described it as a “one stop shop” that relieved her of the burden of trying to coordinate care among multiple services:

“Having one-stop shop for everything—like broken leg, I have an issue, I have one person I can call, I can text her right now… and she will direct me and she will advocate for me…But most families do not have that. They go to the ortho or they go to ENT, and they are all separate entities. In my case, it’s not a separate entity at all. Yes, I go to ortho, I go to ENT, but if I have an issue with either of them, I talk to complex care, and then they’ll sort it out” (Lilly, mother).

The lack of coordination and communication among specialists was also an issue in the school context. Parents described multiple examples of information not being relayed between school staff. For example, one parent shared with her son’s guidance counselor the news about the child’s father passing away, but this information was not relayed to his teachers. Parents noted that recommendations made by school-based therapists were either not acted upon by the child’s teachers, or if they were, they only lasted until the end of the school year and the following year the entire process would have to begin from scratch, in effect delaying crucial supports. They also observed that children’s mental health issues were not recognized nor attended to by school professionals, and advocated for improved training in this area.

Conversely, a few parents in the study brought up positive examples of coordinated and holistic solutions that relieved some of the toll on them. Meenah, for example, described a summer camp for her child that provided all necessary equipment and accessories, down to the labels for their belongings. Kora mentioned a helpful social worker who completed funding applications on her behalf (even writing out her name), and then provided her with an envelope and a stamp.

## Discussion

A key insight derived from this study is the need for people with lived experience—in our case, children and parents—to inform research and policy on issues that are relevant to their lives. Children’s views in particular are underrepresented in research and policy. Eliciting their views may require alternate approaches, such as using creative methods or accepting assistance from children’s desired conversation partners (in our case, parents).

Our conversations with parents and children contributed to numerous insights about their needs moving forward. Like other disastrous events in the past, the Covid pandemic revealed “social conditions that are less visible but nonetheless present in everyday life” (66). This study echoes other existing research on the experiences of disabled children and their families during the pandemic, as outlined in the introduction. For children with disabilities and their families, the pandemic shone a light on the cracks in supports that have always been there (2). For some families, the cracks deepened to the point of becoming sinkholes (2). Some families found unexpected benefits in some of the solutions introduced to cope with pandemic measures, most notably the use of virtual technologies for learning, working, social connections or shopping. However, children’s (and more broadly, families’) ability to participate in community and live meaningful lives was complicated by systemic gaps and failures at all levels and sectors that both predate and continue beyond the immediate pandemic period. The gaps identified in the study include a lack of investment in training and compensation of workers in care-adjacent professions (for example, educational assistants or personal care workers), resulting in low quality and high turnover; excessive bureaucracy and rigidity in education and social programs; a lack of coordination among and within education, healthcare and social service sectors; and an overall lack of consideration of disability rights at all levels. These gaps are typically buffered by parents, who made personal, career and financial sacrifices—but this is not tenable indefinitely. Participants’ accounts illustrated that they need flexible solutions and supports in different sectors; social connections/supports that are constant and reliable; and comprehensive and holistic “wraparound” support for the entire family. The fact that the majority of children in our study were reported to not fit into the existing categories of needs and supports as set by the healthcare, education and social support systems, suggests that these categories do not recognize the complexity and dynamism of real-life health circumstances and needs.

**Figure 8:**
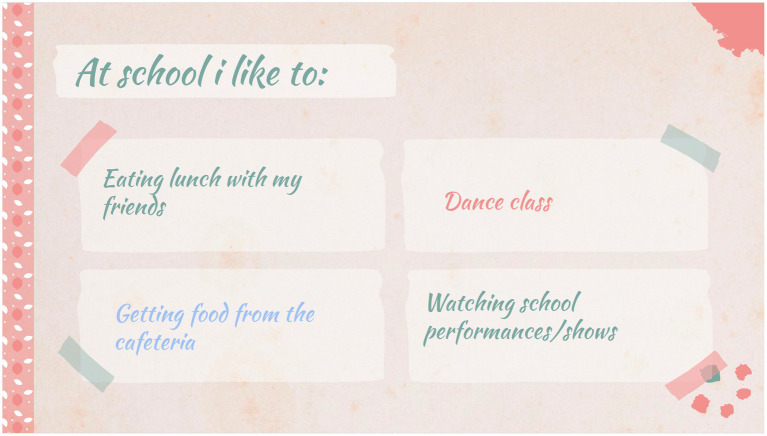
For children, school is about more than education: it offers opportunities to socialize and take part in activities that they enjoy (‘Helen’, 14 y.o.).

Participants’ accounts also highlighted the interconnections of domains of life, as set out in the ICF framework (44). These interconnections need to be recognized by integrated policies and solutions across sectors. For example, school is not just a place where children learn academic content, but also a key site where children grow and develop by making friends, participating in fitness activities, receiving therapeutic supports, and having fun (Figure 8). However, solutions and supports tend to be siloed by sector: for example, school-based therapists are only mandated to work on school-based goals; community health nurses are generally mandated not to provide school-based supports; and recommendations from health professionals (usually in the form of support letters) rarely translate into actions in schools. In fact, several parents told us that “school is the biggest problem.” Therefore, one of the take-aways from this study is that healthcare and education need to coordinate. Both at the school district level and in individual schools and classrooms, educational professionals need to be able to consistently access and collaborate with healthcare professionals who are familiar and have expertise in the child’s disability and subsequent needs (for example, regarding the amount of educational assistant support a child should receive). This finding echoes numerous existing studies in the area of disability and education (67–69), which draw attention to current practices that impede true collaboration between parents, healthcare professionals and educators, and suggest possible strategies for changing them. One possible step toward starting to integrate education and “life” goals could be to replace current individualized education plans with individualized support plans that include “lived health” goals (70).

Many of the components of care desired by parents align with the tenets of Family-Centered Service. These include: holistic approaches that see the “whole family” and the family situation beyond the medical situation; continuity and reliability of services and supports; and supports that are strength-based and non-diagnosis specific (71). Parents also shared the need for improved accessibility of services, in terms of (i) timing (meaning, timely interventions, as opposed to children aging out of services) (ii) geography (some families live hours away from the closest specialist); and (iii) other equity-based considerations, such as the families’ ability to access services hinging on the parents’ ability to advocate for them (which in turn is shaped by factors such as parents’ education and proficiency in English).

Another overarching finding from this study is the vital role of caregiving of children with disabilities as a contribution to the functioning of society. When most of the world shut down during the pandemic, children still needed to be looked after, physically and emotionally. When institutions that traditionally look after some aspects of children’s well-being and development (e.g., schools, children’s rehabilitation clinics, respite programs, recreation programs), went online or closed altogether, parents had no choice but to provide hands-on support in all aspects of life as needed. Although the pandemic constituted an extreme situation, parents’ accounts illustrate that the cracks in the caregiving support network both predated the pandemic and continue to persist in the “normal” times. These cracks speak to the overall low priority that our society places on all forms of caregiving and related institutions that provide care, such as healthcare and education.

In order for children and families to be at their best, the essential value of caregiving needs to be recognized and embedded into policies (2). There are many ways in which this could be done, for example: appropriate staffing, training and compensation for people in care or care-adjacent professions (including nurses, personal support workers and educational assistants) to offset shortages and improve quality and continuity of care; organizational policies in schools and medical/rehabilitation center that prioritize continuity of care (in contrast to simply filling spots), along with transition protocols when staff changes need to occur; employment support for parents/caregivers to attend appointments and therapy sessions (e.g., flexible work arrangements); or sustainable caregiver benefits for parents who provide carework beyond “typical” parenting duties.

### Policy implications

The accounts of the parents in this study show that pockets of “good” (i.e., coordinated, holistic, supportive, and flexible) care do exist, and are possible to implement. Such positive examples can be found across different sectors and include the complex care program at Lilly’s son’s hospital; the summer camp for Meenah’s son; or the skating program for Ruth’s son. The principles and practices that guide such programs can be scaled up and embedded in policies that would allow such solutions to become widespread. Such policies should be integrated across systems and sectors and provide infrastructure for decision-making processes that pertain to the interrelated domains of life including (but not limited to): disability; children; caregiving; education; recreation; and social services. Below we outline 10 recommendations that emerge from our work.

People with lived experience (including disabled individuals, children and youth, and parents/caregivers) should have an active say in shaping policies.
All policies across all sectors should be examined through equity, diversity and inclusion lenses, which should always include a disability lens.
There needs to be a wider variety of recreation and community programs for youth across Canada (especially outside of large cities). These programs need to be adequately staffed to allow for individual assistance and attention as needed.
School boards should facilitate the integration of necessary therapies and care within schools, including those typically provided within communities.
School boards need to provide professional learning and development for school staff on pedagogy and assessment for diverse learning needs (e.g., universal design for learning, differentiated instruction).
(a) Application processes for disability-related supports (e.g., funding, therapies, school-based supports) should be streamlined and coordinated between sectors. (b) Eligibility criteria for services should be needs-based and transdiagnostic.
The size of care-related workforce in both healthcare and education needs to be maintained at appropriate levels to ameliorate existing shortages, and support continuity of care, individual attention and relationship-building. This includes nurses, respite workers, education assistants and therapists.
Work conditions and qualifications for these care-related workers need to be improved through increased training and compensation.
Workplaces need to implement caregiver-friendly work policies. These may include: allowing remote or hybrid work options; allowing time to attend children’s medical appointments; or allowing flexible work hours to accommodate caregiving obligations.
Governments need to put in place financial supports for family caregivers. These may include caregiver allowances/income and tax credits.

The ideas proposed above dovetail with policy recommendations that are currently being articulated by other Canadian organizations working in the spaces of disability (72), children (73), and caregiving (74). We plan to further refine them in the next stages of our work (outlined in the section on Next Steps).

### Strengths and limitations

The majority of parents who took part in our study were mothers (with the exception of one father). Despite our efforts to recruit parents of both sexes (e.g., our recruitment materials contained the gender-neutral language “parents” rather than “mothers”), only one father volunteered to participate in this study. This ratio of mothers to fathers is typical of childhood disability research, where the majority of participants tend to be mothers (75). This means that the parent experiences and perspectives reported in this study are, by and large, those of mothers. It is possible that fathers may have different experiences and different needs, which we were not able to capture. What this study did capture, however, are the experiences of parents who are the primary caregivers of their children and are most knowledgeable about the day-to-day realities and challenges of navigating systems on behalf of their child.

The majority of participants represented in this study were white, university-educated, and spoke English as their first language. Again, this composition reflects a wider trend in childhood disability research, where most participants hail from more privileged social locations in terms of socio-economic background, education and ethnic/racial background (76). These parents were able to access networks and resources that are not available to parents from less privileged demographics (for example, not all parents have the language skills and knowledge of systems to appeal to their provincial human rights commissions). Furthermore, families who experience additional structural disadvantages (e.g., newcomers to Canada, Indigenous or racialized Canadians, families from lower socioeconomic backgrounds) likely face additional challenges and have additional needs that are not captured in this study. These barriers have been reported in the general healthcare literature, and include racism and discrimination on the part of healthcare or education workers; inequitable resource allocation (for example, lack of services in remote Indigenous communities); lack of culturally appropriate services; and language barriers (77, 78). More work is needed to capture the experiences of families from these equity-deserving groups, and our organization is presently working on building relationships with organizations that serve members of these communities to enable us to learn more about their experiences and needs in future studies. However, even parents who are relatively privileged, well-informed, and skilled at navigating systems, nonetheless report many systemic failures, a fact that illustrates how much more work remains to be done to improve those systems for children and families.

Another consideration is that this study is based on a relatively small number of participants. However, the fact that it captures the perspectives of both parents and children is a contribution as this approach is relatively rare in childhood disability research. Furthermore, parents of children of varying ages and diagnoses identified similar issues and needs, which contributes to the theoretical generalizability of the perspectives reported here.

A particular strength of the study is the inclusion of children/youth with disabilities as “junior researchers.” Although we were not able to ensure that the junior researchers represented all possible youth demographics (for example, all of the junior researchers were male), having input from youth on the study design and the interpretation of data enhanced the relevance of the research process and the validity of our findings.

### Next steps

Qualitative findings presented in this article will be used to inform the development of a survey in the next phase of this work. This approach will allow us to verify and generalize these findings with a larger sample size across Canada.

## Conclusion

This study explored the experiences of Canadian children with disabilities/special healthcare needs and their parents throughout the Covid pandemic and the post-pandemic period, to understand what supports and services they need and want moving forward. Families’ experiences were complex and nuanced, but key themes were identified pertaining to needs that cut across various domains of life and are not diagnosis-specific. Namely, families need services that are flexible; oriented around continuity and relationship-building; comprehensive; coordinated across sectors; and accounting for the needs of the whole family. These findings suggest the need to reorient healthcare, education and social support systems from market-based values of efficiency and individualism to a more collectively-based ethic of care.

## Data availability statement

The datasets presented in this article are not readily available because of the need to safeguard participant confidentiality. Requests to access the datasets should be directed to pozniakk@mcmaster.ca.

## Ethics statement

The studies involving humans were approved by Hamilton Integrated Research Ethics Board. The studies were conducted in accordance with the local legislation and institutional requirements. Written informed consent for participation in this study was provided by the participants’ legal guardians/next of kin. Written informed consent was obtained from the individual(s), and minor(s)’ legal guardian/next of kin, for the publication of any potentially identifiable images or data included in this article.

## Author contributions

KP: Conceptualization, Formal analysis, Funding acquisition, Investigation, Methodology, Resources, Writing – original draft, Writing – review & editing. AS: Conceptualization, Data curation, Formal analysis, Investigation, Project administration, Writing – review & editing. GC: Conceptualization, Formal analysis, Writing – review & editing. AD-K: Conceptualization, Formal analysis, Writing – review & editing. DG: Conceptualization, Formal analysis, Writing – review & editing. JL: Conceptualization, Formal analysis, Writing – review & editing. WC: Conceptualization, Formal analysis, Writing – review & editing. CH: Conceptualization, Formal analysis, Writing – review & editing. SP: Conceptualization, Formal analysis, Writing – review & editing. SR: Conceptualization, Formal analysis, Writing – review & editing. JW: Conceptualization, Formal analysis, Writing – review & editing. OC: Conceptualization, Formal analysis, Funding acquisition, Resources, Supervision, Writing – review & editing.
