# Supplementary figures and images for: What supports and services post COVID-19 do children with disabilities and their parents need and want, now and into the future?
Source: Front Public Health. 2024 Apr 8;12:1294340. doi: 10.3389/fpubh.2024.1294340 (PMC11036871; doi:10.3389/fpubh.2024.1294340)

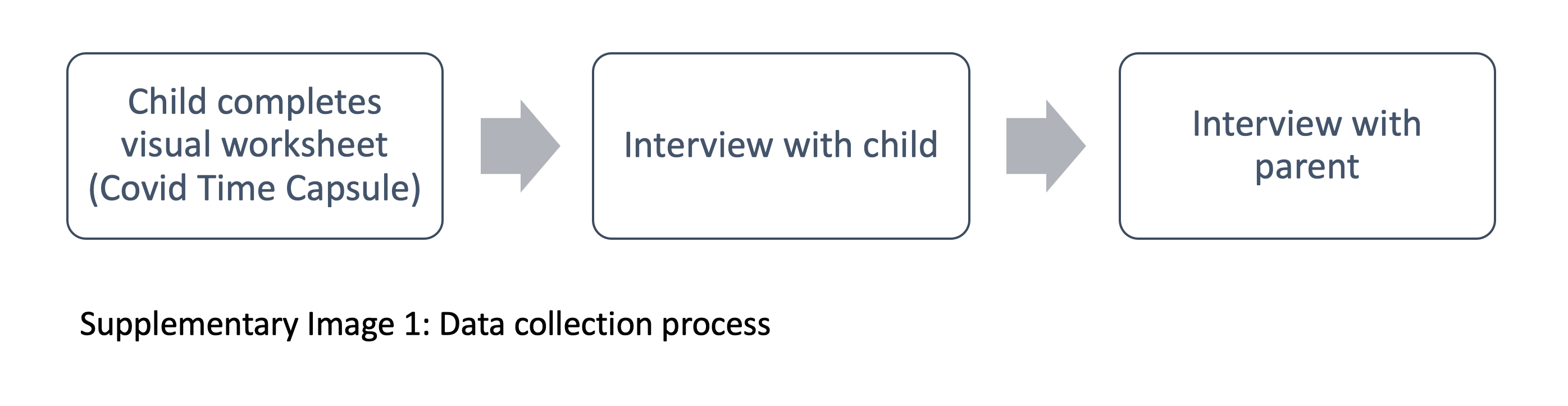

Supplement: Supplementary file 1 [file Data_Sheet_1.docx]
