# Supplementary material for: What supports and services post COVID-19 do children with disabilities and their parents need and want, now and into the future?
Source: Front Public Health. 2024 Apr 8;12:1294340. doi: 10.3389/fpubh.2024.1294340 (PMC11036871; doi:10.3389/fpubh.2024.1294340)

MY COVID-19 TIME CAPSULE

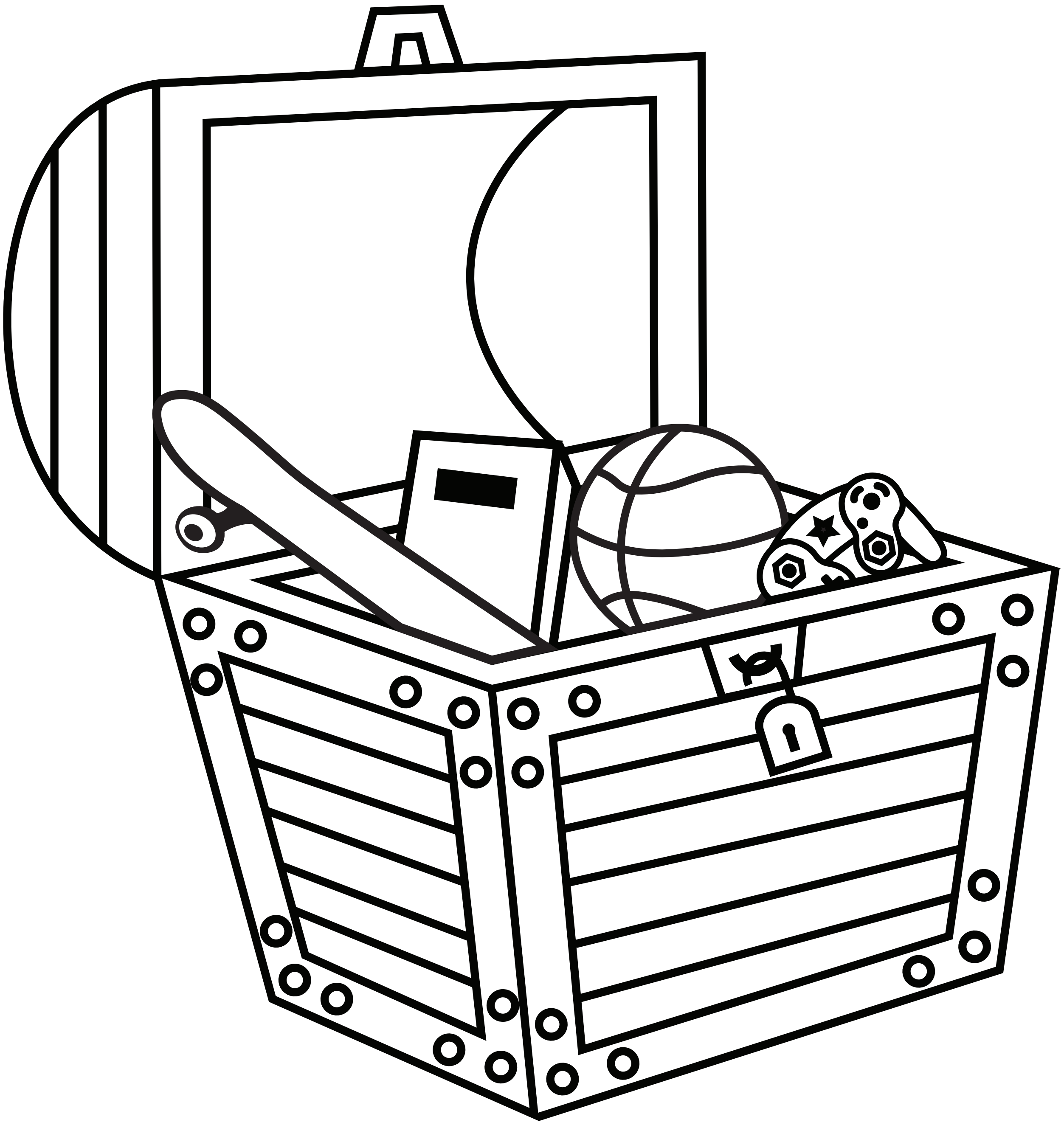

Participant ID: ____________________

ABOUT ME

I am____________ years old.

I live in___________________.

This is me!

My favourite animal is....

My favourite colour is....

My favourite type of music is....

My favourite things are....

MY FAMILY

You can add pictures, draw, or write about your family.

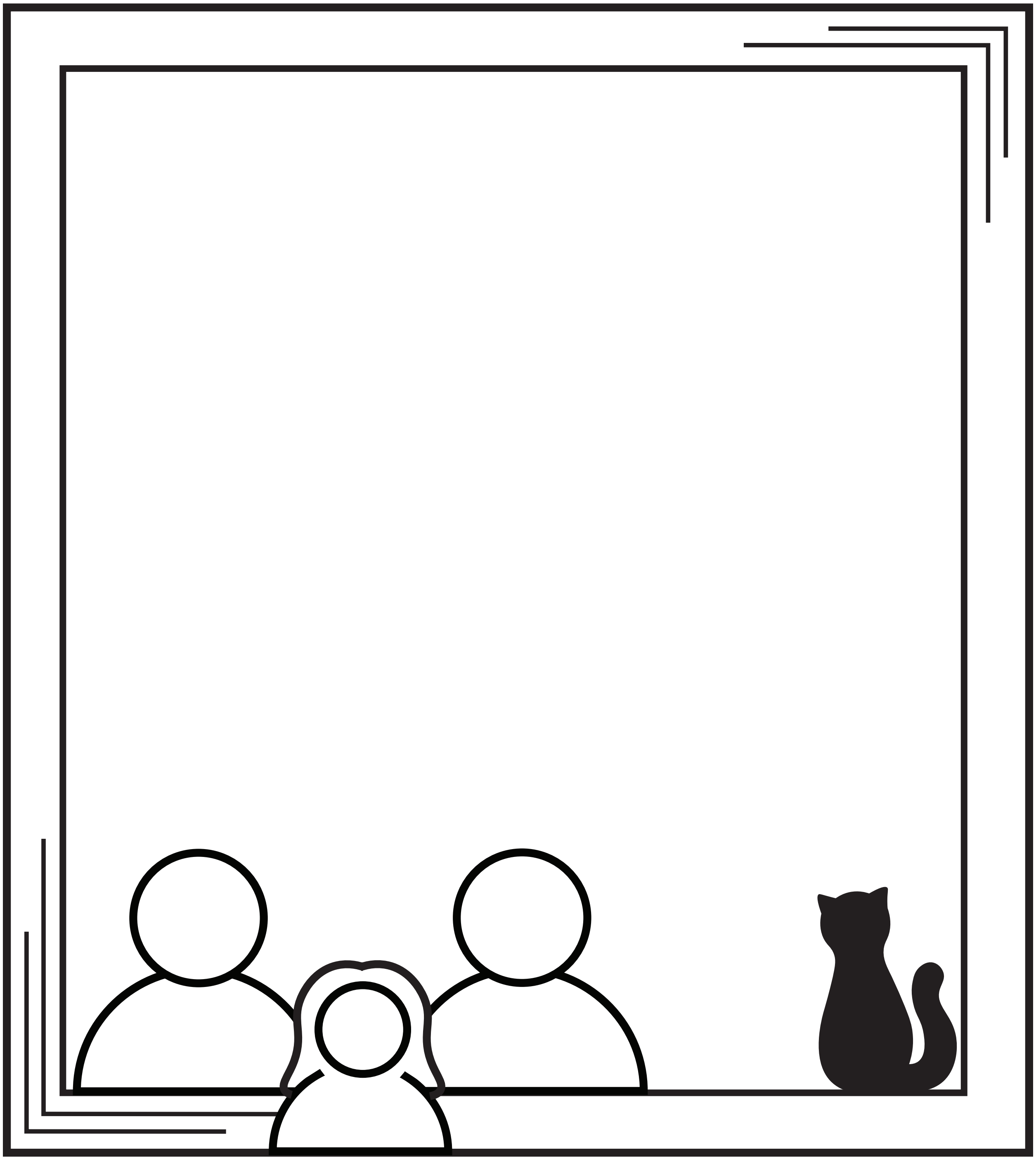

FRIENDS, HELPERS, AND SPECIAL PEOPLE.

You can add pictures, draw, or write about your friends, helpers, and the special people in your life.

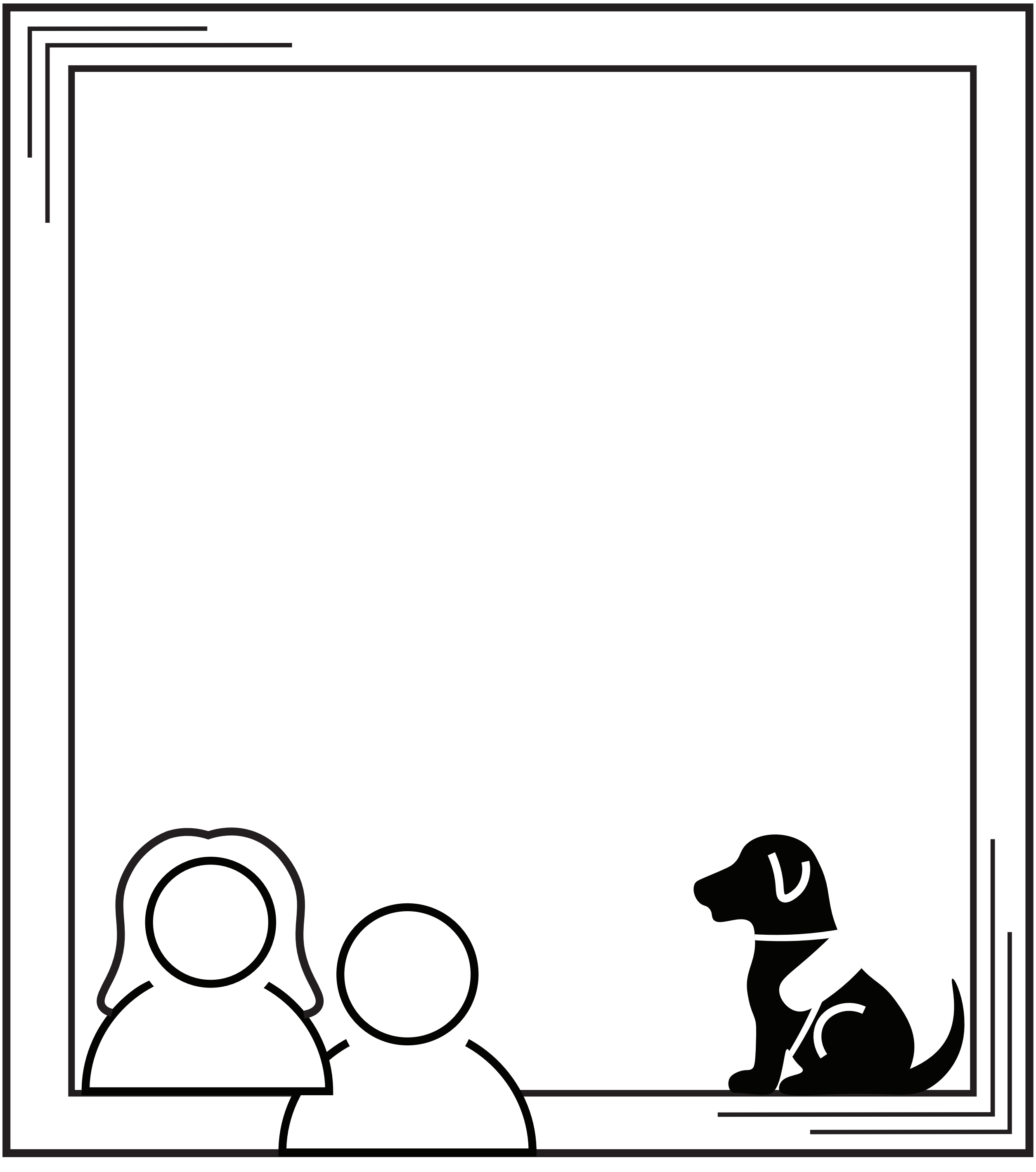

DURING THE SCHOOL DAY I LIKE TO...

You can add pictures, draw, or write about what you like to do at school.

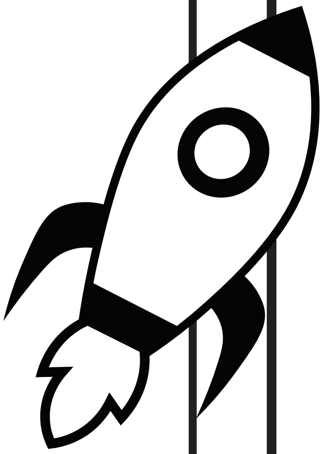

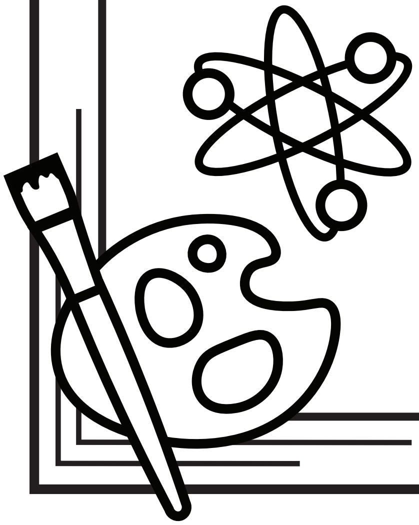

THESE ARE THE THINGS I LIKE TO DO TO MOVE MY BODY.

You can add pictures, draw, or write about about the things you like to do to move your body.

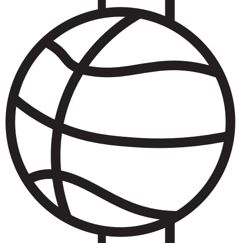

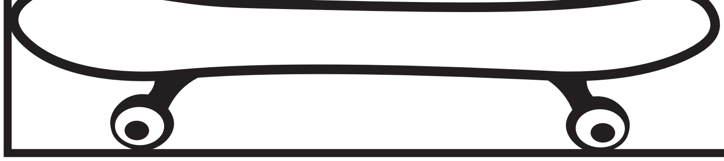

THINGS I DO FOR FUN...

You can add pictures, draw, or write
about the things you like to do to for fun.

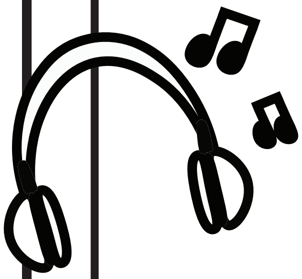

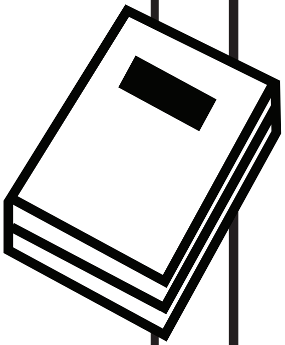

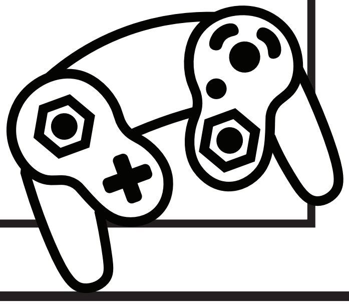

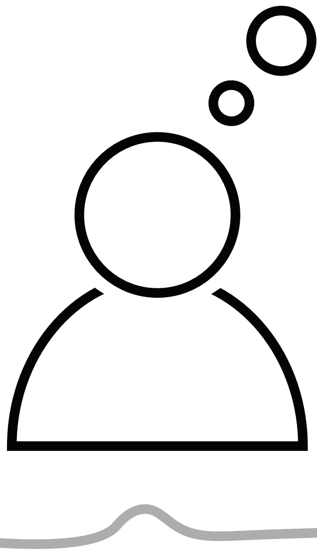

I LOOK FORWARD TO...

You can add pictures, draw, or write about something you look forward to doing in the future.

You can add pictures, draw, or write about
anything else you want to add in your time capsule.

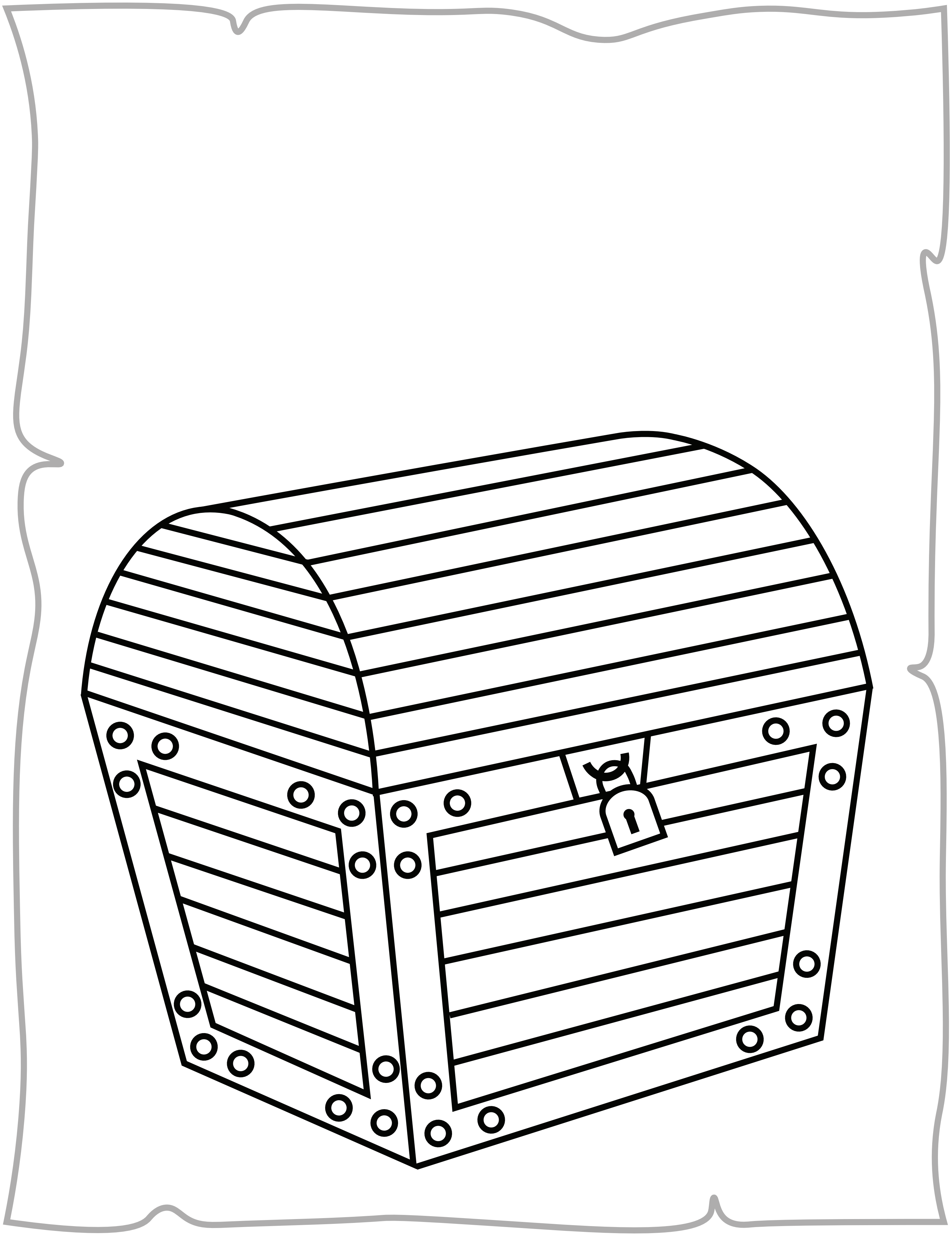

Supplement: Supplementary file 2 [file Data_Sheet_2.pdf]
